# The minimum energy required to build a cell

**DOI:** 10.1038/s41598-024-54303-6

**Published:** 2024-03-04

**Authors:** Edwin Ortega-Arzola, Peter M. Higgins, Charles S. Cockell

**Affiliations:** 1 UK Centre for Astrobiology, School of Physics and Astronomy, University of Edinburgh, Edinburgh, UK; 2 Department of Earth Sciences, University of Toronto, Toronto, ON Canada

**Keywords:** Gibbs energy, Biosynthesis, Virtual cell, Group Contribution Algorithm (GCA), Biogeochemistry, Bioinformatics, Biological models, Astrobiology, Metabolomics, Cellular microbiology, Bioenergetics, Biogeochemistry, Bioinformatics, Biological models, Astrobiology, Metabolomics, Cellular microbiology, Bioenergetics

## Abstract

Understanding the energy requirements for cell synthesis accurately and comprehensively has been a longstanding challenge. We introduce a computational model that estimates the minimum energy necessary to build any cell from its constituent parts. This method combines omics and internal cell compositions from various sources to calculate the Gibbs Free Energy of biosynthesis independently of specific metabolic pathways. Our public tool, Synercell, can be used with other models for minumum species-specific energy estimations in any well-sequenced species. The energy for synthesising the genome, transcriptome, proteome, and lipid bilayer of four cell types: *Escherichia coli*, *Saccharomyces cerevisiae*, an average mammalian cell and JCVI-syn3A were estimated. Their modelled minimum synthesis energies at 298 K were $9.54\times 10^{-11}$ J/cell, $4.99\times 10^{-9}$ J/cell, $3.71\times 10^{-7}$ J/cell and $3.69\times 10^{-12}$ respectively. Gram-for-gram synthesis of lipid bilayers requires the most energy, followed by the proteome, genome, and transcriptome. The average per gram cost of biomass synthesis is in the 300s of J/g for all four cells. Implications for the generalisability of cell construction and applications to biogeosciences, cellular biology, biotechnology, and astrobiology are discussed.

## Introduction

Liquid water is one of the key requirements for life because it is the only known solvent that allows biochemical reactions to occur^1^. However, for an aqueous system to be habitable, the energy available to an organism must equal or exceed the energetic costs associated with its maintenance^2,3^, among other requirements^1^. Life utilises different energy sources, commonly involving chemical redox and/or radiation pathways, the yield of which is then used to build and/or repair biomass^2–4^. Precisely how much energy is required to synthesise this biomass is highly organism- and environment-dependent, and an open and crucial question is how to generalise this energetic cost across cell types. Existing models have estimated the energy required for biomass synthesis by calculating ATP (Adenosine tri-phosphate is the energy ‘currency’ used by many forms of life) requirements of specific synthesis pathways^5^, generalising across species via energy consumption in steady-state ecosystems^6,7^, or by approximating the energy requirement as that to build only proteins^8^. However, with the increasing abundance and availability of omics data, it should be possible, by extending this latter technique, to estimate the minimum energy required to build all the biomacromolecules of a single cell of any sequenced species. Such a technique will advance these existing methods by providing fast environment- and organism-specific minimal energy requirements to build biomass for growth or maintenance with applications across the biogeosciences and biotechnology.

Biosynthesis is intrinsically linked to metabolic pathways as an energy uptake process. While specific pathways vary among organisms based on their context, the laws of thermodynamics can provide a universal framework to understand the minimum energy required for reactions that convert precursor molecules, or building blocks, into biomolecules^8–13^. The minimum energy obtained by the change in Gibbs free energy, $\Delta G_r$, can help reveal metabolic pathways that are efficient enough to use this amount of energy^14,15^.

This calculation process requires the standard Gibbs free energy of formation ($\Delta G_f^{\circ }$) for the compounds involved and the cell composition. However, the thermodynamic data set available for complex bio-polymers is relatively sparse. One way to make the most of the available data is to utilise a group contribution algorithm (GCA). This method estimates $\Delta G_f^{\circ }$ for large molecules by approximating it as the sum of the $\Delta G_f^{\circ }$ of its constituent parts^13,16–18^. GCAs have been previously used to compute the standard molar thermodynamic properties of unfolded proteins at elevated temperatures and pressure^4,19^, to estimate minimal costs of biosynthesis in extraterrestrial environments^20,21^, and to calculate the energetic cost of synthesising the building blocks of biomolecules from inorganic compounds in extreme environments—such as oxic and anoxic deep-sea hydrothermal systems^6,8,22^.

In this work, we present a model that leverages the ongoing explosion in omics data availability to extend such calculations, assembling all of the biochemical building blocks of a cell into biomacromolecules. This includes not just proteins but also DNA, RNA, phospholipids and carbohydrates. This approach is independent of specific metabolic pathways, which vary from organism to organism, and variation in the chemical environment. This independence from conventional metabolic considerations represents considerable advantages over traditional approaches. Specifically, it can be generalised for settings where the full microbial community and associated biogeochemistry is incomplete or yet to be defined, or be applied to investigate the prospective habitability space for any well-sequenced species under the in situ thermodynamic conditions in more detail than was previously possible. This model, therefore, can serve as a means to sidestep frequently encountered bottlenecks in fields where considerable biouncertainty exists, such as cellular biology, biotechnology, the biogeosciences and, in particular, astrobiology.

Here we examine the case studies of *Escherichia coli* (*E. coli*), *Saccharomyces cerevisiae* (*S. cerevisiae*), an average mammalian cell, and JCVI-syn3A. The JCVI-syn3A cell is a synthetic organism characterised by its minimalistic genome, transcriptome, and proteome^23,24^. It represents a streamlined cellular model with the bare minimum genetic and proteomic content required for life. This minimalistic design makes JCVI-syn3A an ideal subject for examining the fundamental lower energy boundaries required for cellular synthesis. This approach is especially crucial in fields such as astrobiology, where understanding the minimal energetic thresholds for life is essential in exploring the potential habitability of extraterrestrial environments and the prospects for synthetic life forms.

## Results

### The minimum energy necessary to build a cell

**Figure 1:**
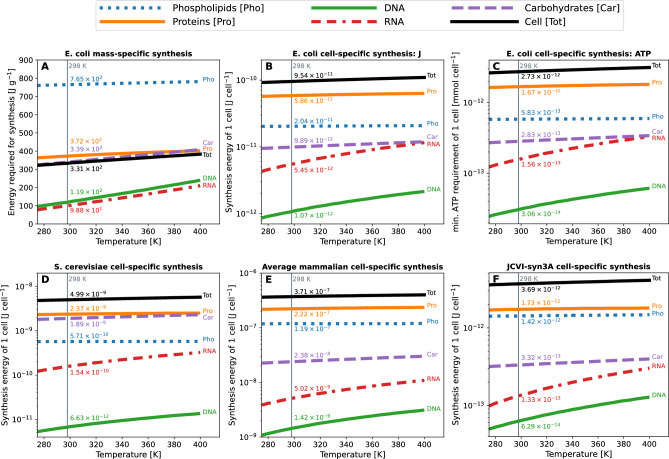
Comparative analysis of minimum energetic costs for cellular synthesis across different organisms at different temperatures calculated using synercell. The panels display the energy cost (horizontal axes) vs. temperature (vertical axes). Values noted below are at 298 K: (**A**) ***E. coli*** **Mass-Specific Synthesis** Displays the energetic cost in Joules per gram for *E. coli*’s DNA, RNA, proteins, and phospholipids. The energy to synthesise one gram of *E. coli* cells is 331 Joules. (**B**) ***E. coli*** **Cell-Specific Synthesis** Shows the synthesis energy of one *E. coli* cell, including the contributions from its genome, transcriptome, proteome, and lipid bilayer. The energy necessary to synthesise one *E. coli* cell is $9.54\times 10^{-11}$ J. (**C**) ***E. coli*** approximate millimolar amount of ATP required for syntheses in Panel B, assuming 35,000 J per mole of ATP. (**D**) ***S. cerevisiae*** **Cell-Specific Synthesis** The energetic costs in building *S. cerevisiae* and its cellular components. The energy necessary to synthesise one cell is $4.99\times 10^{-9}$ J. (**E**) **Average Mammalian Cell Synthesis** The energetic costs in building an average mammalian cell and its cellular components. The energy necessary to synthesise one average mammalian cell is $3.71\times 10^{-7}$ J. (**F**) **JCVI-syn3A Cell Synthesis** The energetic costs in building a JCVI-syn3A cell and its cellular components. The energy necessary to synthesise one JCVI-syn3A cell is $3.69\times 10^{-12}$ J. Cell compositions represent averaged values derived from various sources (Supplementary Table S1), aiming to capture a general representation across diverse growth phases. The average composition represents a generalised perspective of cell energetics, mainly reflecting conditions akin to the natural environmental lag phase.

**Figure 2:**
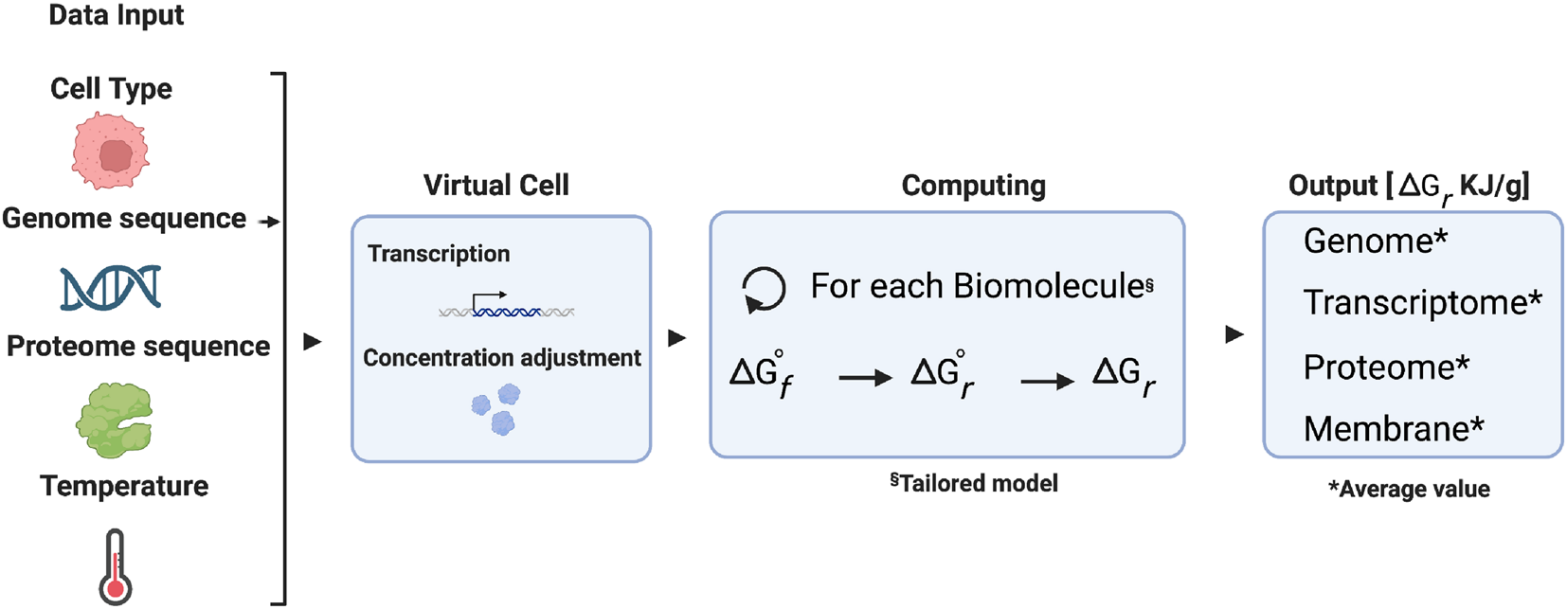
Workflow summary of Synercell. The tool will first require data input, including the type of cell (bacterial, yeast, mammalian or JCVI-syn3A), a genome sequence, a protein sequence and the temperature at which the user wants to calculate the energy. The tool creates a virtual cell with the omics data input, transcribing the genome and adjusting the concentration pool according to the cell type. Using the GCA, the tool uses tailored models for each biomolecule type to estimate its $\Delta G_f^{\circ }$ at the chosen temperature. Next, the tool obtains the $\Delta G_r^{\circ }$ following its stoichiometry. Finally, it calculates $\Delta G_r$ combining experimental and theoretical concentrations data for each constituent.

The minimum energy needed to build a cell as defined here is the sum of the energy required to assemble all its components into their biomolecules. Based on the cell composition and biomolecule structure, we tailored group-contribution models which estimate the energy required to build a cell’s genome, proteome, transcriptome and lipid bilayer (See “Methods” section). These algorithms build a virtual cell by reading a DNA and protein sequence associated with the cell type. A visual representation of the results can be found in Fig. 1, cell-specific values for *E. coli* in Table 1 and a comparison with other cell types and studies in Table 2. For the present study, these calculations were made at temperatures from 275 to 400 K for four different model cells: *E. coli* (Table 1), *S. cerevisiae*, an average mammalian cell and JCVI-syn3A (Table 2). At 298 K, the energy required to synthesise one single *E. coli* cell is $9.54\times 10^{-11}$ J/cell (331 J/g) and $3.69\times 10^{-12}$ J/cell (329 J/g) for JCVI-syn3A. For *S. cerevisiae* and a mammalian cell, the energy required is $5\times 10^{-9}$ J/cell (311 J/g) and $3.71\times 10^{-7}$ J/cell (354 J/g), respectively. A summary of the algorithm’s workflow can be found in Fig. 2 and the units of the calculations in Table 3.

**Table 1:** *E. coli* composition and the energy necessary to synthesise its components at 298 K.

Cell component	Amount (g/100 g Cells)	Energy per gram (kJ/g)	Energy per cell (J/Cell)	Energy fraction
Genome	3.13	0.12	$1.07\times 10^{-12}$	1.1%
Transcriptome	19.25	0.10	$5.54\times 10^{-12}$	5.7%
Proteome	55	0.37	$5.86\times 10^{-11}$	60.8%
Lipid bilayer	9.31	0.79	$2.099\times 10^{-11}$	21.8%
Carbohydrates*	10.21	0.34	$1.009\times 10^{-11}$	10.4%
Metabolites and ions	3.10	–	–	0.0%
One *E. coli* cell		1.73	$9.54\times 10^{-11}$	

The per gram values in this table are not adjusted to the respective fraction of mass.

*The Carbohydrates’ energy was calculated from the average energy value of the computed biomolecules in order to adjust the energy necessary to synthesise one full cell or one gram of cells. (See Supplementary information). We assume that any energy required to synthesise metabolites and ions is negligible.

**Table 2:** Cell synthesis cost comparison in J (g cell$$)^{-1}$$ and ATP mmol (g cell)$$^{-1}$$ at 298 K. See footnotes for details on unit conversion.

	Joules (g cell$$)^{-1}$$						
	This work				Lynch and Marinov^33,34^	McCollom and Amend^6^	Stouthamer^5^^e^
	Mammalian	*S. cerevisiae*	JCVI-Syn3A	*E. coli*	*E. coli*	*E. coli (Anoxic)*	*E. coli*
DNA	1.37	0.42	5.61	3.77	90.01	542.00	20.16
RNA	4.84	9.7	11.85	19.37	867.24		80.50
Proteins	211.59	147.13	154.82	204.63	4322.50	690.00	669.90
Phospholipids	113.01	35.48	127.21	73.31	2157.92	89.00	4.90
Carbohydrates	22.77	117.86	29.62	29.60	0.00	83.00	71.82
Cell	**353.58**	**310.59**	**329.11**	**330.67**	**7437.67**	**1404.00**	**847.28**

	ATP mmol (g cell)$^{-1}$						
	This work				Lynch and Marinov^33,34^	McCollom and Amend^6^^f^	Stouthamer^5^
	Mammalian	*S. cerevisiae*	JCVI-Syn3A	*E. coli*	*E. coli*	*E. coli (Anoxic)*	*E. coli*
DNA	0.04	0.01	0.16	0.10	2.57^a^	15.49	0.58
RNA	0.14	0.28	0.34	0.53	24.78^b^		2.30
Proteins	6.05	4.27	4.42	5.58	123.50^c^	19.71	19.14
Phospholipids	3.23	1.03	3.63	2.00	61.65^d^	2.54	0.14
Carbohydrates	0.65	3.42	0.85	0.96	0.00	2.37	2.05
Cell	**10.11**	**9.02**	**9.40**	**9.17**	**212.50**	**40.11**	**24.21**

Significant values are in bold.

The per gram values in this table, are adjusted to their respective fraction of mass.

^a^The genome energy was calculated per Ref.^33^ considering a genome size of 4,608,319 bp^35^ and a cost of 101 ATP per bp^33^.

^b^The transcriptome’s energy was calculated per Ref.^33^ considering a cost of 46 ATP per nucleotide^33^, an average RNA size of 1000 nts^36^ and 9.73$\times 10^{4}$ RNAs.

^c^The proteome’s energy was calculated per Ref.^33^ considering 2.68$\times 10^{6}$ proteins with an average of 320 amino acids^37^ and an average cost of 26 ATPs per AA^38^.

^d^The phospholipids energy was calculated per Ref.^33^ considering a surface area of 4.42 $\mu \text {m}^2$^39^ and an average cost of glycerophospholipids of 367 ATP molecules^34^ for a total of 1.72$\times 10^{10}$ molecules of ATP. We also calculated the number of phospholipids based on the mass fraction of dry weight (9.3%) and the average molecular weight of a phospholipid (740 Da) for a total of 4.45$\times 10^{10}$ molecules of ATP. The value in the table is the average between both calculations.The values of ATP were obtained assuming that each molecule of ATP yields 35 kJ/mol.

^e^Stouthamer 1975 reported values in mol ATP (g cell)$^{-1}$. Conversion to J (g cell)$^{-1}$ was done assuming 1 mole of ATP yields 35 kJ, as above.

^f^McCollom & Amend 2005 reported values in J (g cell)$^{-1}$. Conversion to mmol ATP (g cell)$^{-1}$ was done assuming 1 mole of ATP yields 35 kJ, as above.

**Table 3:** Thermodynamic, input and output data parameters and units used in our models.

Thermodynamic	Symbol	Unit
Standard Gibbs Free Energy of Formation	$\Delta G_f^{\circ }$	kJ mol
Standard Gibbs Free Energy of Reaction	$\Delta G_r^{\circ }$	kJ mol
Temperature	T	K
Reactant concentration	[R]	mol/L
Product concentration	[P]	g/cell
Cellular Gibbs Free Energy of synthesis	$\Delta G_r$	J/cell
		ATP/ (g cells)
Input data		
DNA sequence		Bp
Protein sequence		AA
Biomolecule concentration		g/cell
Ouput data		
Gibbs Free Energy	$\Delta G_r$	J/g

Preliminary results indicate, as has been noted previously^25^, that minimal energy expenditure in life generally scales with mass by (1) the different contributions of the different cell constituents and (2) different concentrations of the metabolites. However, the synthesis cost of a gram of biomass of each of the four species is remarkably similar indicating a consistent fundamental floor in per-gram cost of biomass synthesis. On a per gram basis, synthesising DNA at 298 K requires 0.12 kJ/g, which is higher than the 0.10 kJ/g needed for RNA in *E. coli*. However, when we consider the cell’s mass fraction, RNA, which constitutes a larger fraction of mass, has a higher net energy requirement when building each cell (0.019 kJ/ g cells) compared to DNA (0.0037 kJ/ g cells), accounting for 5.7$\%$ and 1.1$\%$, respectively, as detailed in Table 1. On the other hand, despite the lipid bilayer accounting for only 9$\%$ of the cell’s mass fraction, it is the second most energy-intensive component, requiring 21$\%$ of the cell’s total synthesis energy ($2.099\times ~10^{-11}$ J/cell), as shown in Table 1. Table 2 also shows estimates of the energetic cost of biomass synthesis from other studies. Our results are lower, suggesting we have identified a thermondynamic minimum, but there are important caveats to consider when comparing these estimates (“Discussion” section).

For all models at all temperatures, we estimated $\Delta G_f^{\circ }$ for the respective biomolecules, which allowed us to obtain the standard Gibbs free energy of reaction $\Delta G_r^{\circ }$. We computed the mean molar Gibbs free energy ($\Delta G_r$; Eq. (1)), correcting for the absolute intracellular concentrations of products and reactants reported in different studies (Supplementary Tables S1–S4)^26,27^. The results suggest that the higher the temperature, the more expensive it is to synthesise a cell regardless of the organism Fig. 1, although fully testing this universality would require repeated calculations over many more organisms and variations in internal cell composition with temperature. The energetic requirements across the temperature scale (275–400 K) vary by approximately 16$\%$ for *E. coli* ($9.20\times 10^{-11}$ J/cell to $1.097\times 10^{-10}$ J/cell), 15$\%$ for *S. cerevisiae* ($4.87\times 10^{-9}$ J/cell to $5.73\times 10^{-9}$ J/cell), 8.8$\%$ ($3.65\times 10^{-7}$ J/cell to $4\times 10^{-7}$ J/cell) for an average mammalian cell and 12% ($3.58\times 10^{-12}$ J/cell to $4.1\times 10^{-12}$ J/cell) for JCVI-syn3A. This temperature range was chosen to cover known non-freezing habitable temperatures for low salinity fluids. The current maximum temperature observed for life is 395 K^28^.

### The minimum energy necessary to build a proteome

We used the GCA approach described in Higgins and Cockell^4^ and Amend and Hegelson^19^ to calculate the formation energy of every protein in the cell. The amino acid composition can be obtained from an input sequence for any organism. However, we do not consider the concentration of each protein in the cell and instead use an average value for proteins, consistent with these previous studies. To calculate the energy required to synthesise the proteome of a single cell, we first multiply this value by the fraction of the cell’s dry mass made up by the proteome (which for *E. coli* is 0.55^22,29^). We then divide the resulting energetic fraction by the number of cells ($3.51\times 10^{12}$) in 1 g. The energy needed to synthesise the proteome of one *E. coli* cell is $5.86\times 10^{-11}$ J at 298 K or 204.67 J per g of cells as seen in Tables 1 and 2, respectively. The energy necessary to assemble the proteome of *S. cerevisiae* is $2.37\times 10^{-9}$, for an average mammalian cell is $2.24\times 10^{-7}$ and for JCVI-syn3A is $1.73\times 10^{-12}$.

The molar Gibbs free energy of synthesising one gram of proteins remains similar throughout the temperature scale (0.37 kJ/g at 275 K and 0.40 kJ/g at 400 K), varying only 10$\%$. Of all the biomolecular components, the $\Delta G_r$ needed to synthesise proteins is the second most stable throughout the temperature scale after the the lipid bilayer, which varies by only 3$\%$. The mean $\Delta G_r$ for protein polymerisation is of a similar order of magnitude to other estimates ($\sim$0.5 kJ/g from Higgins and Cockell^4^; 0.347 kJ/g from Amend et al.^8^).

### The minimum energy necessary to build a genome and a transcriptome

We extended the approach used for the proteome in this work and other previous studies^6,13,19,19,22,30^ to calculate the energy required to synthesise a genome and transcriptome, thus capturing a larger and more representative percentage of the total cell mass. Details of the calculations can be seen in the “Methods” section.

The $\Delta G_r$ needed to synthesise the genome and transcriptome at 298 K is $1.06\times 10^{-12}$ J and $5.44\times 10^{-12}$ J respectively for an *E. coli* cell (Table 1), $6.71\times 10^{-12}$ and $1.56\times 10^{-10}$ for *S. cerevisiae*, $1.44\times 10^{-9}$ and $5.08\times 10^{-9}$ for a mammalian cell and $6.28\times 10^{-14}$ and $1.32\times 10^{-13}$ for JCVI-syn3A. Compared to proteins, the energetic variability caused by temperature is greater for the transcriptome and genome throughout our temperature scale, estimating that the transcriptome becomes 63$\%$ more expensive to synthesise at 400 K ($1.15\times 10^{-11}$ kJ/g) than at 275 K ($4.29\times 10^{-12}$ kJ/g) and the genome by 60$\%$ ($2.15\times 10^{-12}$ kJ/g vs $8.6\times 10^{-13}$ kJ/g) for *E. coli*.

Because the energetic contribution of intramolecular bonds can significantly influence a biomolecule’s overall $\Delta G_f^{\circ }$, we also employed the GCA to calculate the energy requirement of the critical ester bond involved in DNA binding. Removing the standard free energy of formation of the ribose and a phosphate from ribose-5-phosphate gives a value of 23.14 kJ/mol (Supplementary Fig. S2) which approximately matches the hydrolysis value obtained experimentally by Dickson et al.^31^ ($-22.175$ kJ/mol) at 25 C and pH 7.

### The minimum energy necessary to build a lipid bilayer

We developed a straightforward model to calculate the energy of a cell lipid bilayer composed entirely of palmitoyl-2-oleoyl-sn-glycero-3-phosphocholine (POPC). This model was created with the assistance of the GCA and is based on the direct assembly of its building blocks^32^ and a balanced reaction from metabolites available in the SUPCRT slop07 database (Table S4). As seen in Fig. 1, the energy to synthesise one gram of POPC at 298 K is 0.764 kJ/g, making it the most expensive component of the cell. However, this might be due to the simplicity of this methodology compared to the model for the proteome, genome and transcriptome. For an *E. coli*-sized cell, the energy required is $2.099\times 10^{-11}$ J, $5.71\times 10^{-10}$ J (Table 1) for a *S. cerevisiae*-sized cell, $1.18\times 10^{-7}$ J for a mammalian-sized cell and $1.42\times 10^{-12}$ for JCVI-syn3A (Fig. 1).

This value is the most stable of all biomolecules considered throughout the temperature range, being only 3$\%$ more expensive to synthesise a lipid bilayer at 400 K ($2.08\times 10^{-11}$ J) than at 275 K ($2.025\times 10^{-11}$ J) for *E. coli*. Despite methodological differences, our value (73.31 J/(g cell)) is similar to that calculated by McCollom and Amend^6^ (89 J/(g cell) in anoxic conditions) as seen in Table 2. See Supplementary Document SD2 for the whole data set at different temperature points.

### The minimum energy necessary to build Carbohydrates

Finally, it is worth noting that developing a universal thermodynamic model for carbohydrate synthesis is a significant challenge due to the inherent structural diversity and isomerism found in carbohydrates. The limited experimental data for complex polysaccharides adds to this challenge; as such, we used the mean from the other biomolecule models to simplify the calculations to 1 g as disclosed in Table 1.

## Discussion

Establishing a standardised methodology to calculate the minimum energetic requirements for cellular biosynthesis that defines the most efficient metabolic pathways across diverse cell types can provide insights into the energetic constraints of life in different environments and guide research in astrobiology, cellular biology, and biotechnology. This work offers a comprehensive, data-driven approach to elucidate the minimum energetic requirements of cellular biosynthesis. The model calculates organism and environment-specific energy requirements, elaborating on other approaches which rely on model organisms, highly specific applications, or generalising across microbial communities. We have shown that per gram of dry weight, mammalian cells, *S. cereviscea*, *E. coli*, and even the ‘minmial cell’ JCVI-syn3 have similar minimum energetic costs of biosynthesis.

It is important to note that the “energy required for cellular synthesis” and the “minimum energy necessary for cellular synthesis” should be interpreted differently. The former refers to the typical energy expended in-practice, accounting for specific metabolic pathways, environmental influences and biological inefficiencies. The latter, this study’s focus, represents optimal conditions, yielding the lowest possible energy required to build a cell. While the in-practice energy reflects typical cellular operations and can be influenced by possible metabolic heterotrophic inefficiencies or the availability of partially constructed carbon sources, the minimum energy is a foundational, agnostic reference for highly efficient metabolisms. One method of quantifying the efficiency of metabolism is by calculating a Gibbs energy dissipation rate (in kJ (g cells)$^{-1}$ h$^{-1}$). This paramaterises the energy which is not utilized in metabolism, and is lost as entropy, heat, or through other inefficiencies. It varies with growth rate and appears to plateau at high growth rates^40^. As Synercell is integrated into microbial growth models, it may be used in the future to examine how much dissipation is caused by the difference between the two synthesis energies described above.

Historical research on biosynthesis has predominantly centred on the cell maintenance energy, biomass synthesis energy from metabolic models^41^, exploring ATP requirements for specific synthesis pathways^5^, energy dynamics within chemolithotrophic communities^6^, or generalising biomass synthesis from inorganic precursors based on fixed stoichiometries^7^. The approach presented here instead provides insights into a lower thermodynamic floor of energy required to build a cell, which could shed light on the ultimate biophysical limit of efficiency for microbial growth. This approach uses a wider variety of omics data than those listed above. It plays a vital role in lending specificity and variability to the biomolecules under consideration. The variablity in composition and size given by input sequences is adjusted to fix reactants and products’ concentration pools. Thus, the model can be deployed for any well-sequenced species, yielding cell-specific biosynthesis energy requirements for application in biogeosciences, cellular biology, biotechnology, or astrobiology.

Our minimum energy necessary for cellular synthesis can be tentatively compared with other estimates that used the various approaches above. Some comparisons for *E. coli* are listed in Table 2. The synthesis energies computed in this work for *E. coli* are significantly lower than these other estimates. This owes to the methodological differences between the studies, and our goal in this work to find a fundamental thermodynamic minimum. The largest difference is between this work and the estimates from Lynch and Marinov^33,34^. For each cell component, these estimates are $\sim$20–40 times larger than our predictions. This most likely owes to the Lynch and Marinov estimates including smaller building blocks than Synercell and that model’s association with empirical data. For example, the majority of the Lynch and Marinov^33^ synthesis energy ATP cost is associated with building nucleotides with polymerisation a minor (of order percent) contributor^33^, whereas Synercell focuses on building the helix structure. This likely accounts for one portion of the discrepancy, with the remainder associated with alternative inefficiencies such as the residual energy loss described above. McCollom and Amend^6^ suggest that the actual observed energy expended on growth processes is approximately an order of magnitude larger than thermodynamic cost of synthesising the constituent building blocks, and their column in Table 2 only characterises that process, not polymerisation. If the McCollom and Amend^6^ column reflects the building block synthesis then, and Synercell represents the polymerisation cost, the remainder between the sum of these and the Lynch and Marinov^33,34^ column may represent the cellular inefficiency in biomass production in nature. Higgins and Cockell^4^ calculated that, for proteins at $\approx$25 $^\circ$C, the synthesis of amino acids from organic precursors and the cost of polymerisation are approximately 700 and 500 J (g proteins)$^{-1}$ respectively. However, this begins to diverge with elevating temperature and amino acid synthesis becomes more energy intensive ($\approx$4 times more expensive at 100 $^\circ$C)^4^. The Synercell proteome polymerisation estimate for *E. coli* at 25 $^\circ$C is 372 J (g proteins)$^{-1}$, in broad agreement with Higgins and Cockell ($\approx$500 J (g proteins)$^{-1}$)^4^ and Amend et al. (347 J (g proteins)$^{-1}$)^8^. To our knowledge, this is the first application of a GCA for DNA, RNA, and phospholipid polymerisation so it is difficult to verify these results against other studies. Similar relationships are observed between the different components and the other studies noted above and in Table 2.

In Table 2 synthesis energies are also presented in mmoles of ATP per gram of cells in order to compare with other empirically validated results^33,34^. However, the ATP energy yield is influenced by internal cell concentrations and physicochemical parameters like temperature and pressure, which can vary significantly among different organisms^20^ and even within the same organism under different growth states. Consequently, while comparing ATP costs is a prevalent approach in the literature, this method only sometimes provides a straightforward comparison due to these variable internal and environmental factors. Our analysis, therefore, treats these ATP cost estimations as part of a broader, context-dependent framework rather than as absolute values for direct comparison. As such, conversion between the units of the studies in Table 2 may account for some of the discrepancy in energy synthesis values.

Our approach to estimating the minimal energy requirements for cell synthesis is an alternative to using biomass compositions derived from flux balance analysis (FBA). FBA, a well-recognised method for studying metabolic networks, often involves challenges in accurately capturing the stoichiometry of biomass reactions, a point highlighted by recent studies^42,43^. These challenges stem from the difficulty in obtaining detailed experimental data for all major biomass components, compounded by the variability and complexity of metabolic networks. To sidestep the inherent uncertainty in the biomass reaction stoichiometry used in FBA models, we instead introduce variability in size and composition by reading input sequences. While FBA models are critical for understanding cellular metabolism, they often focus on the growth-associated maintenance (GAM) demand of ATP, making it hard to understand the minimum energy necessary to synthesise these components. Reported values for *E. coli* cell synthesis calculated with FAB include 23 mmol (g cells)$^{-1}$^44^, 59.81 mmol (g cells)$^{-1}$^41^, 53.81 mmol (g cells)$^{-1}$^45^ and 75.38 mmol (g cells)$^{-1}$^46^, which are larger than our estimates, and, as above, this difference likely characterised cellular inefficiencies and complexities such as GAM and energy dissipation. In contrast, Synercell aims to provide an energetic baseline while staying flexible to any cell with known genome and proteome sequences. This approach is particularly advantageous for analysing cells with less characterised metabolic networks, where detailed experimental data for biomass composition are unavailable.

Results for the JCVI-syn3A cell model can also be compared to some other calculations, albeit in a more limited way than *E. coli*. JCVI-syn3A is an interesting case study, because it was engineered to function as a ‘minimal cell’^24^. This makes it an ideal example to probe the fundamental minimal energy necessary to synthesise a cell. On a per-cell basis, the minimum synthesis energy of JCVI-syn3 was the lowest amongst our sample of four—but it was also the smallest cell so that result alone is limited. On a per-gram basis, all four organisms examined in this work have a similar minimum synthesis cost, and any differences are likely caused primarily by differences in internal cell composition, and secondarily by genome and proteome complexity. Breuer et al.^24^ provides some estimates of the ATP requirement to synthesise JCVI-syn3A DNA, RNA, and proteins—0.24, 0.14, 21.2 mmol ATP respectively—but those are based on *E. coli*-like synthesis costs so direct applicability to this organism is limited. Synercell results were generated with the JCVI-syn3A internal metabolites composition, and genome and proteome sequences.

In this work, we have expanded the scope of existing methodologies for peptide synthesis^19,20^ to include the energy calculations for a cell’s DNA, RNA, and lipid content. This was not possible for carbohydrates owing to the extensive diversity in carbohydrate structures, their varied functional roles across different organisms, lack of standardised structural description^47^ and the limited availability of thermodynamic data. The vast heterogeneity in carbohydrates implies that no single structure can adequately capture the essence of all cell types. Instead, we adopted an alternative strategy where we adopted an average value approach for the carbohydrate content, as has been previously done for all non-proteome components^4,8,19^. This approach allowed us to integrate carbohydrates into our whole-cell calculations, ensuring a more comprehensive and representative model, albeit with an acknowledgement of the simplifications necessitated by the complexity of carbohydrate diversity.

Furthermore, our model employs POPC as a representative phospholipid to approximate the energetic costs associated with membrane synthesis. While POPC is a prevalent component in many cell types, this membrane simplification poses limitations in fully capturing the energetic nuances associated with synthesising more complex cell membranes. Cell membranes comprise a rich mixture of various lipid species and proteins and in this model the latter part is calculated as part of the proteome algorithm. Approximately 30% of proteins in a cell are in the membrane^48^. To get a closer approximation to the membrane value we need to consider that the energetic cost of this protein component would be approximately 30 % of the proteome’s value (58.57 J/(g cells) or 1.76$\times 10^{-11}$ J/ cell for *E. coli*). The lipid bilayer cost is 69.95 J/(g cells) or 2.10$\times 10^{-11}$ J/cell, giving a total of 128.53 J/(g cells) or 3.86$\times 10^{-11}$ J/cell for an *E. coli* membrane (values from Table 2). Table 2 also summarises similar estimates of the cell constituents of *E. coli* from other studies using slightly different methods and chemical environments.

Our model stands out due to its adaptability. It can be refined with additional thermodynamics and omics, allowing for species-specific energy estimates. Conversely, since our model’s input requires biomolecule sequences to perform the calculations, it can only perform DNA, RNA and protein calculations based on omics data. Despite previous efforts to sequence phospholipids and carbohydrates^47,49^, there is still a lack of standardised methodologies and data for these biomolecules. Therefore, our model only includes one ‘hand-made’ generic model per biomolecule type. Consequently, since accurately calculating the minimum energy needed to synthesise a cell requires more thermodynamic information for phospholipids and carbohydrates, we provide an open-source tool for different applications that can be updated as data becomes available.

In the development of this model, we evaluated two key aspects: (1) the accuracy of the GCA in constructing a biomolecule’s $\Delta G_f^{\circ }$, and (2) the use of different $\Delta G_f^{\circ }$ standards for the building blocks (Table 4). First, we built the nucleotides in two ways: phosphate + deoxyribose + adenine (block method 1) and phosphate + deoxyadenosine (block method 2) (Supplementary Fig. S1). We obtained similar results when comparing the $\Delta G_r$ obtained with the different methodologies and the standard nucleotide’s $\Delta G_f^{\circ }$ from the SUPCRT slop07 database^50^. Furthermore, we tested this method for chemical bonds and validated the results with experimental data (Supplementary Fig. S2), indicating this is a reliable method. Secondly, we examined thermodynamic and biological standards $\Delta G_f^{\circ }$ for the building blocks^18^ to ensure consistency in results. Each standard estimated the same $\Delta G_r$, likely due to the lack of H^+^ in the overall reaction and our assumption that ionic strength is close to zero^20^. Furthermore, although the only physicochemical parameter considered here was temperature, the models could be corrected for chemical differences by considering the corresponding change in cellular content, if any. Our models show a broad floor when compared to results from other studies which examine a variety of chemical environments^4–6,19^.

In the future, our model could benefit from integrating more variables. For example, variations in internal pH among organisms can influence cellular composition stability^51,52^. Environmental shifts can also affect energy consumption in biomacromolecule synthesis. Higgins and Cockell^4^ showed that rising temperatures make amino acid synthesis a leading energy expense in protein formation. Additionally, McCollom and Amend^6^ found that anaerobic conditions are more conducive to building block synthesis than aerobic ones due to specific oxidation states. In anaerobic settings, the altered oxidation state affects the concentrations of crucial dissolved compounds, influencing biomolecule synthesis. Moreover, we have utilised cell compositions from diverse sources to approximate an average value, aiming to represent a broad spectrum of growth phases. While this approach provides a broad overview, future studies may benefit from analysing cell composition in specific growth phases to assess dynamic changes in energetic costs and maintenance requirements. This would enhance the granularity of our analysis and allow us to examine how changes in the absolute internal cell composition—both reactants and products—impact the overall energetic cost of cell synthesis.

When the model is deployed for analyses of microbial communities in situ, local instantaneous geochemical data should be leveraged to correct internal cell concentrations and their effect on the present biosynthesis calculations, unlocking faster and more robust biomass turnover calculations than are currently possible^20^. Typically, the biological data which serves as input parameters for microbial models are inferred from culture-based studies which themselves are controlled and well-defined but may be time consuming to perform. The model presented here only depends on the omics data of any given organism and its internal composition, so only requires the latter to be updated using insights into the local geochemistry to generate site-specific energetic requirements of biomass synthesis. This could additionally be extended for analyses of habitability and growth through deep time, and to model how the energy requirement changes with its environment^53^.

This study’s primary goal was determining the minimal energy necessary to assemble a cell, a key metric for understanding the basal energy requirements essential for life. The energetic requirement of biomass synthesis is a critical component of bioenergetic habitability models^4^ and a controlling parameter in estimates of biomass turnover, which are pertinent to biosignature production and, by extension, constraining the feasibility of life detection on other worlds^20^. Additionally, our findings have significant implications in biotechnology, offering a pathway to optimise energy efficiency in microbial production systems and synthetic biology applications. By establishing a benchmark for the minimum energy needed to construct cellular biomass, our model, Synercell, is a tool for identifying and enhancing energy-efficient pathways in various biotechnological processes^54–57^. The potential integration of our model, Synercell, with other predictive models, (e.g., amide bond synthesis^58^), can enhance the accuracy of bioenergetic predictions across diverse environmental conditions.

In conclusion, this study introduces a comprehensive, data-driven model to understand the minimum energy requirements for cellular biosynthesis. It is a valuable tool for cellular biology, biotechnology, biogeosciences, and astrobiology and can be incorporated into other models. We anticipate its flexibility will encourage further research and data collection, particularly for thermodynamic data related to organisms other than those studied here, and their constituent biomolecules. Ultimately, our research contributes to understanding the energy constraints of life and the factors influencing the fundamental thermodynamic minimum energy requirements for cell construction. This understanding is crucial for exploring life’s boundaries in extreme environments, optimising biotechnological processes, and probing the potential for life beyond Earth.

## Methods

The Gibbs Free Energy (Eq. 1) represents the energy available to do work. By quantifying the Gibbs Free Energy of synthesis for proteins, DNA, RNA, and lipids, we can determine the work required to build these cellular components from their most direct building blocks under given conditions. This value reflects the energetic investment to maintain and/or replicate a cell and offers insights into the efficiency of cellular processes. To calculate the energy for each cellular component, we developed a model tailored to each biomolecule to calculate $\Delta G_f^{\circ }$ and then use internal cell concentrations to calculate the molar Gibbs energy ($\Delta G_r$) of each biomolecule type:

1 $$\begin{aligned} \Delta G_r = \Delta G_r^{\circ } + RT \ln {Q} \end{aligned}$$

where $\Delta G_r^{\circ }$ is the standard reaction Gibbs energy obtained from an average of every biomolecule on the cell, *R* is the ideal gas constant, *T* is the absolute temperature in kelvin, and $\ln {Q}$ is the natural logarithm of the reaction quotient between building blocks (reactants) and biomolecules (products). This equation is used for each value produced at different temperatures (Fig. 2).

### Cell synthesis energy

The total energy to synthesise a cell ($\Delta G_{[synthesis]}$) is the sum of the energy required to synthesise its components—the proteome, genome, transcriptome, and other cellular biomolecules. Numerous approaches to calculating the maintenance energy have been proposed^4,59,60^, and typically depend upon some energy requirement of biomass synthesis for replacement. This work aims to compute synthesis or growth energy, using the Gibbs free energy to synthesise a cell $\Delta G_ {[synthesis]}$ (Eq. 2) from the building blocks of different biomolecule types. For this, we can break down a cell into:

2 $$\begin{aligned} \Delta \mathbf {G_{[synthesis]}} = \Delta G_{[proteome]}+ \Delta G_{[genome]} +\Delta G_{[transcriptome]}+ \Delta G_{[...ome]} \end{aligned}$$

This equation encapsulates the comprehensive energy requirement for cell construction where subscripts refer to the molecule types being synthesised.

### Energy for maintenance and growth

Broadly speaking, organisms use their energy supply for either growth or maintenance^2^ (Eq. 3). Growth processes depend on the energetic cost of biosynthesis (e.g., building proteins or DNA), whereas maintenance processes include all those which consume energy but are not necessarily related to growth^2,22^. Complete cellular energetic calculations can be performed when we gather enough information to calculate the energy to build all the structures of a cell with the cell simultaneously remaining viable^22^:

3 $$\begin{aligned} \Delta \mathbf {G_{[cell]}} = \Delta G_{[synthesis]}+\Delta G_{[maintenance]} \end{aligned}$$

This formula provides a view of the cell’s energy budget, incorporating both the energy for biosynthesis (building biomolecules like proteins and DNA) and maintenance (energy-consuming processes that do not directly contribute to growth).

## Model implementation

Because $\Delta G_f^{\circ }$ for large biomolecules is hard to find in the literature, we made an estimation using the group contribution algorithm (GCA) as similarly done by Mavrovouniotis^16,61^. We calculated the $\Delta G_f^{\circ }$ of proteins, DNA, RNA and a lipid bilayer by summing together the $\Delta G_f^{\circ }$ of their respective building blocks (e.g. amino acids, nucleotides, phospholipids, etc.)^13^. The flowchart in Fig. 2 summarises the procedure of Synercell ’s modules.

The modules obtain the different compounds’ $\Delta G_f^{\circ }$ used for the GCA from the slop07 database and SUPCRT92^50^. This software package calculates the standard molar thermodynamic properties of minerals, gases, aqueous species, and reactions from 1 to 5000 bar and 0–1000 $^\circ$C^50^. The data was accessed and calculated using the reaktoro package for chemical systems, using an implementation of the revised HKF equations^62,63^. Once the $\Delta G_f^{\circ }$ of each biomolecule is obtained, the Gibbs reaction energy ($\Delta G_r^{\circ }$) is obtained with the following:

4 $$\begin{aligned} \Delta G_r^{\circ }=\sum \Delta G_f^{\circ }(\text { products })-\sum \Delta G_f^{\circ }(\text { reactants }) \end{aligned}$$

where *R* is the ideal gas constant (8.314 J/mol K), *T* is the absolute temperature (K), and $\ln {Q}$ is the natural logarithm of the reaction quotient. Because $\Delta G_r$ calculations heavily rely on the absolute concentrations within a cell, we used the building blocks’ (reactant) concentrations obtained experimentally by Bennet et al.^26^ and Park et al.^27^, and biomolecules’ (product) concentrations estimated on the fraction of dry mass in the cell Table S1. For the latter, the variability is adjusted with the help of the input sequences. For instance, if the input data contains five protein sequences, the program will obtain an average from those five protein sequences and adjust it to the final protein mass depending on the cell type.

## Energy to synthesise the proteins

Synpro is the model within the program that implements the GCA approach from Higgins and Cockell^4^ and Amend and Helgeson^19^ to calculate the formation energy of a protein according to its amino acid composition. The $\Delta G_f^{\circ }$ and $\Delta G_r^{\circ }$of a protein are calculated with:

5 $$\begin{aligned} \begin{aligned} \Delta G_{f[ \text{ Protein } ]}^{\circ } =&\Delta G_{f[\textrm{AABB}]}^{\circ }+\left( N_{\textrm{AA}}-N_{\textrm{Gly}}-1\right) \Delta G_{f[\textrm{PBB}]}^{\circ }&+\sum _{i=1}^{19} m_i \Delta G_{f\left[ R_i\right] }^{\circ }+N_{\textrm{Gly}} \Delta G_{f[\textrm{Gly}]}^{\circ } \end{aligned} \end{aligned}$$

where AABB represents the amino acid backbone ($H_2N-CH-COOH$), $N_{AA}$ is the number of amino acids in the protein, $N_{Gly}$ number of Glycines, PBB is the protein backbone ($HN-CH-C=0$) and R is the non-glycine functional group of each amino acid. $m_i$ acts as a counter for the number of occurrences of each non-GLY amino acid *i* in the chain such that $\sum ^{19}_{i=1} {m_i} = N_{AA}$^4^.

Equation (4) is then used to calculate the Gibbs reaction energy ($\Delta G_r^{\circ }$):

6 $$\begin{aligned} \begin{aligned} \Delta G_{r[ \text{ Protein } ]}^{\circ } =&(n-1) \Delta G_{f\left[ \textrm{H}_2 \textrm{O}\right] }^{\circ }+\Delta G_{f[ \text{ Protein } ]}^{\circ }&-\sum _{i=1}^{20} n_i \Delta G_{f\left[ A A_i\right] }^{\circ } \end{aligned} \end{aligned}$$

Synpro calculates the $\Delta G_f^{\circ }$ and $\Delta G_r^{\circ }$ of each protein within the input sequence. Next, the algorithm obtains an average $\Delta G_r^{\circ }$ to represent all the proteins in the proteome sequence. This average value is then used to calculate $\Delta G_r$ taking into account the internal absolute amino acids composition and total protein concentration reported per cell type.

## Energy to synthesise the nucleic acids

Syngen calculates the energy necessary to synthesise DNA and RNA based on a similar approach used in Synpro. Respecting its stoichiometry, we obtained the formation energy of a nucleic acid chain ($\Delta G_{f\left[ N A_{\text{ chain } }\right] }^{\circ }$) using the GCA, which can be summarised with:

7 $$\begin{aligned} \begin{aligned} \Delta G_{f\left[ N A_{\text{ chain } }\right] }^{\circ } =&\left( N_n-1\right) \left\{ \Delta G_{f[ \text{ Ester } \text{ bond } ]}^{\circ }-\Delta G_{f[O H]}^{\circ }\right. \}\ {}&+\sum _{i=1}^4 m_i \Delta G_{f\left[ \text{ Nucleotide } \text{ ion } { }_i\right] }^{\circ } \end{aligned} \end{aligned}$$

We obtained $\Delta G_r^{\circ }$ with:

8 $$\begin{aligned} \begin{aligned} \Delta G_{r\left[ N A_{\text{ chain } }\right] }^{\circ } =&\left( N_n-1\right) \ \Delta G_{f\left[ \textrm{H}_2 \textrm{O}\right] }^{\circ } +\Delta G_{f\left[ N A_{\text{ chain } }\right] }^{\circ }&-\sum _{i=1}^4 n_i \Delta G_{f\left[ \text{ Nucleotide } { }_i\right] }^{\circ } \end{aligned} \end{aligned}$$

The model calculates the energy for the double strand genome according to the input sequence. In parallel, the tool transcribes the sequence by analysing available open reading frames (ORFs) that the program detects between the first available start codon (ATG) and the immediate next stop codon (either TAA, TAG, TGA in this order) transcribing each ORF until the end of the sequence without considering transcription factors or other transcription criteria other than the length of the potential transcript.

## Energy to synthesise the phospholipids

This model assumes all cell membranes are made of phosphatidylcholine (POPC), one of the most abundant phospholipids across cell types^32,64–66^, arranged in a lipid bilayer that does not take into account proteins as the membrane proteins are considered within the proteome calculations. We obtained the $\Delta G_f^{\circ }$ of POPC adding up the formation energies of its building blocks:

9 $$\begin{aligned} \begin{aligned} \Delta G_{f[ \text{ POPC } ]}^{\circ }\quad = \Delta G_{f[P i]}^{\circ }+\Delta G_{f[ \text{ Choline } ]}^{\circ }+\Delta G_{f[ \text{ Glycerol } ]}^{\circ }+\Delta G_{f[ \text{ Palmitate } ]}^{\circ } + \Delta G_{f[ \text{ Oleate } ]}^{\circ }- 4 \Delta G_{f\left[ \textrm{H}_2 \textrm{O}\right] }^{\circ }\ \end{aligned} \end{aligned}$$

Due to the lack of thermodynamic data available for this biomolecule, we used a different approach to calculate $\Delta G_r^{\circ }$ from metabolites as seen in Henry et al.^67^ and Jankowski et al.^68^. The $\Delta G_f^{\circ }$ of the metabolites involved were available in the slop07 database and used to balance a theoretical condensation reaction to estimate the reaction energy:

10 $$\begin{aligned} \begin{aligned} \Delta G_{r[ \text{ lipid } \text{ bilayer } ]}^{\circ }\quad =\quad&N_{P L}\left[ \ \Delta G_{f[P O P C]}^{\circ }+\Delta G_{f[A D P]}^{\circ }+\right. \left. \Delta G_{f\left[ H_2 O\right] }^{\circ }+\Delta G_{f[C O 2]}^{\circ }\right]&\\&-N_{P L}\left[ \Delta G_{f[ \text{ Glucose } ]}^{\circ }+\Delta G_{f[ \text{ Serine } ]}^{\circ }+\Delta G_{f[A T P]}^{\circ }+\Delta G_{f[ \text{ Pyruvate } ]}^{\circ }+\Delta G_{f[ \text{ Malonate } ])}^{\circ } \right]&\end{aligned} \end{aligned}$$

$N_{PL}$ is the number of phospholipids in the lipid bilayer estimated by comparing the weight of POPC and the weight of the lipids in a cell. $\Delta G_r$ was calculated using internal compositions of the metabolites The stoichiometry of this reaction can be found in the Supplementary material Table S4.

## Assessment and validation of the GCA

To test the accuracy of the GCA up to the nucleotide scale, we broke down the nucleotides of *E. coli*’s genome in three different ways when calculating the cost of synthesis (Supplementary Fig. S1). Using thermodynamic values available in the slop07^69^ database for the different building blocks, we calculated the energy to synthesise the *E. coli* genome. Despite minor discrepancies between the two alternative methods (0.12 kJ/g vs. 0.152 kJ/g), results were generally consistent. The synthesis energy for dAMP varied by about 10 kJ/mol between methods, hinting at potential intramolecular interactions. Additionally, the energy requirements for critical DNA-binding bonds, such as the ester bond in ribose-5-phosphate, resolved by this testing closely matched experimental values (approx. 22.17 kJ/mol)^31^. These results reinforce the reliability of our approach for determining the $\Delta G^{\circ }_f$ of nucleotides.

## Additional notes and standardisation

The thermodynamic standard for $\Delta G_f^{\circ }$ used in this work should not be confused with the alternative biological standard $\Delta G_f^{`\circ }$. The thermodynamic standard represents conditions at pH value of 0 (i.e., a concentration of H^+^ equal to 1 M) and ionic strength of zero. In biological standard conditions, pH is set to 7 and ionic strength usually 0.1, but is often less rigorously defined (Table 4). Standards are converted to actual molal quantities as outlined above.

**Table 4:** Conditions for each type of standard condition. The (thermodynamic) standard conditions refer to those typically used in chemistry and physics while the biological recreates those found in typical intracellular physiological environments.

	Standard ($\Delta G^{\circ }$)	Biological ($\Delta G^{`\circ }$)
Pressure	1 bar	1 bar
pH	–	7
Ionic strength	0 M	0.1 M
Net charge	–	Variable
Temperature	298K	Variable
Reactant concentrations	1 mol	Variable

In this study, we used values for *E. coli* at 37 $^\circ$C in aerobic glucose containing minimal medium at a doubling time of 40 min and Values for *S. cerevisiae* grown at 30 $^\circ$C in aerobic 0.5% glucose containing minimal medium at a doubling time of 160 min. Values represent an average of reported values for various growth conditions. Energies of all building blocks used in our calculations, derived from thermodynamic and biological standards, are sourced from the SUPCRT92 slop07 database^50^. This is a comprehensive repository for these critical thermodynamic data, ensuring the accuracy and consistency of our thermodynamic calculations across the temperature range studied.

### Supplementary Information

Supplementary Information 1.

Supplementary Information 2.

## Data Availability

The data used in this study are classified as follows: (A) Input data: (1) *E. coli* str. K-12 substr. MG1655, complete Genome (GenBank: U00096.3) and Proteome (UniProt ID: UP000000625). (2) *S. cerevisiae* Strain: S288C. Genome (RefSeq:GCF 000146045.2) and Proteome (UniProt ID: UP000002311). (3) Homo Sapiens reference Genome (Sequence GRCh37) and Proteome (UniProt ID UP000005640). (4) The fraction of Mass data for each cell type is disclosed in the supplementary document. (5) The thermodynamic dataset used for calculating the Gibbs free energy of formation of large biomolecules was derived from the SUPCRT slop07 dataset^50^. This publicly available dataset can be accessed through 10.1016/0098-3004(92)90029-Q. (6) The dataset containing internal cell concentrations for various cell types was sourced from the study by Bennet et al.^26^. This dataset is publicly available and can be accessed through the 10.1038/nchembio.186. (B) Models: (1) Synercell can be accessed to calculate the energy of the genome, transcriptome, proteome and cell lipid bilayer in the GitHub repository of Synercell. As new data becomes available, new dictionaries can be added for different cell types. (C) **Output data** (1) The dataset generated in this study includes the Gibbs Energy of Reaction for each biomolecule, adjusted to its internal concentration at different temperatures. This dataset is available in the Supplementary material.

## References

[1] Cockell, C. S. *et al.* Habitability: A review. *Astrobiology* **16**, 89–117 (2016). 26741054 10.1089/ast.2015.1295

[2] Hoehler, T. An energy balance concept for habitability. *Astrobiology* **7**, 824–838 (2007). 18163865 10.1089/ast.2006.0095

[3] Hoehler, T. M., Bains, W., Davila, A., Parenteau, M. N. & Pohorille, A. Life’s requirements, habitability, and biological potential. in (Meadows, V. S., Des Marais, D. J., Arney, G. N. & Schmidt, B. E. Eds.) *Planet. Astrobiol*. 37–69 (University of Arizona Press, 2020).

[4] Higgins, P. M. & Cockell, C. S. A bioenergetic model to predict habitability, biomass and biosignatures in astrobiology and extreme conditions. *J. R. Soc. Interface* **17**, 20200588 (2020). 33081642 10.1098/rsif.2020.0588 PMC7653372

[5] Stouthamer, A. H. A theoretical study on the amount of ATP required for synthesis of microbial cell material. *Antonie van Leeuwenhoek* **39**, 545–565 (1973). 4148026 10.1007/BF02578899

[6] McCollom, T. & Amend, J. A thermodynamic assessment of energy requirements for biomass synthesis by chemolithoautotrophic micro-organisms in oxic and anoxic environments. *Geobiology* **3**, 135–144 (2005). 10.1111/j.1472-4669.2005.00045.x

[7] Kleerebezem, R. & Van Loosdrecht, M. C. A generalized method for thermodynamic state analysis of environmental systems. *Crit. Rev. Environ. Sci. Technol.* **40**, 1–54 (2010). 10.1080/10643380802000974

[8] Amend, J., LaRowe, D., McCollom, T. & Shock, E. The energetics of organic synthesis inside and outside the cell. *Philos. Trans. R Soc. B Biol. Sci.* **368**, 20120255 (2013). 10.1098/rstb.2012.0255 PMC3685458 23754809

[9] Schrödinger, E. *What is Life? The Physical Aspect of the Living Cell* (Cambridge University Press, 1944).

[10] Asimov, I. *Life and energy* (Avon, 1977). OCLC: 898831983.

[11] Schneider, E. & Kay, J. Life as a manifestation of the second law of thermodynamics. *Math. Comput. Model* **19**, 25–48 (1994). 10.1016/0895-7177(94)90188-0

[12] Krebs, H., Kornberg, H. & Burton, K. A survey of the energy transformations in living matter. *Ergeb. Physiol.* **49**, 212–298 (1957). 13609573 10.1007/BF02269485

[13] LaRowe, D. & Dick, J. Calculation of the standard molal thermodynamic properties of crystalline peptides. *Geochim. Cosmochim Acta* **80**, 70–91 (2012). 10.1016/j.gca.2011.11.041

[14] Greinert, T., Vogel, K., Maskow, T. & Held, C. New thermodynamic activity-based approach allows predicting the feasibility of glycolysis. *Sci. Rep.* **11**, 1–9 (2021). 33731762 10.1038/s41598-021-85594-8 PMC7971085

[15] Stettner, A. I. & Segrè, D. The cost of efficiency in energy metabolism. *Proc. Natl. Acad. Sci.* **110**, 9629–9630 (2013). 23729810 10.1073/pnas.1307485110 PMC3683743

[16] Mavrovouniotis, M. L. Group contributions for estimating standard gibbs energies of formation of biochemical compounds in aqueous solution. *Biotechnol. Bioeng.* **36**, 1070–1082 (1990). 18595046 10.1002/bit.260361013

[17] Dick, J., LaRowe, D. & Helgeson, H. Group additivity calculation of the standard molal thermodynamic properties of aqueous amino acids, polypeptides and unfolded proteins as a function of temperature, pressure and ionization state. *Biogeosci. Discuss* **2**, 1515–1615 (2005).

[18] Noor, E., Haraldsdóttir, H., Milo, R. & Fleming, R. Consistent estimation of gibbs energy using component contributions. *PLOS Comput. Biol.* **9**, e1003098 (2013). 23874165 10.1371/journal.pcbi.1003098 PMC3708888

[19] Amend, J. & Helgeson, H. Calculation of the standard molal thermodynamic properties of aqueous biomolecules at elevated temperatures and pressures II. Unfolded proteins. *Biophys. Chem.* **84**, 105–136 (2000). 10796027 10.1016/S0301-4622(00)00116-2

[20] Higgins, P. M. *“Modelling extraterrestrial habitability, biomass and biosignatures through the bioenergetic lens”*. Ph.D. thesis, University of Edinburgh, Edinburgh, UK (2022).

[21] Higgins, P. M., Glein, C. R. & Cockell, C. S. Instantaneous habitable windows in the parameter space of Enceladus’ Ocean. *J. Geophys. Res. Planets*. **126**, e2021JE006951 (2021).

[22] LaRowe, D. & Amend, J. The energetics of anabolism in natural settings. *ISME J.* **10**, 1285–1295 (2016). 26859771 10.1038/ismej.2015.227 PMC5029197

[23] Hutchison, C. A. *et al.* Design and synthesis of a minimal bacterial genome. *Science* **351**, aad6253. 10.1126/science.aad6253 (2024). Publisher: American Association for the Advancement of Science. 10.1126/science.aad6253 27013737

[24] Breuer, M. *et al.* Essential metabolism for a minimal cell. *eLife* **8**, e36842. 10.7554/eLife.36842 (2019). Publisher: eLife Sciences Publications, Ltd. 10.7554/eLife.36842 PMC6609329 30657448

[25] Hoehler, T. M. *et al.* The metabolic rate of the biosphere and its components. *Proc. Natl. Acad. Sci.* **120**, e2303764120 (2023). 37307462 10.1073/pnas.2303764120 PMC10288578

[26] Bennett, B. *et al.* Absolute metabolite concentrations and implied enzyme active site occupancy in Escherichia coli. *Nat. Chem. Biol.* **5**, 593–599 (2009). 19561621 10.1038/nchembio.186 PMC2754216

[27] Park, J., Rubin, S., Xu, Y.-F., Amador-Noguez, D. & Shlomi, F. Metabolite concentrations, fluxes, and free energies imply efficient enzyme usage. *Nat. Chem. Biol.* **12**, 482–489 (2016). 27159581 10.1038/nchembio.2077 PMC4912430

[28] Takai, K. *et al.* Cell proliferation at 122 C and isotopically heavy CH4 production by a hyperthermophilic methanogen under high-pressure cultivation. *Proc. Natl. Acad. Sci.* **105**, 10949–10954 (2008). 18664583 10.1073/pnas.0712334105 PMC2490668

[29] Battley, E. H. Calculation of the heat of growth of Escherichia coli k-12 on succinic acid. *Biotechnol. Bioeng.* **37**, 334–343 (1991). 18597375 10.1002/bit.260370407

[30] Amend, J. & Helgeson, H. Calculation of the standard molal thermodynamic properties of aqueous biomolecules at elevated temperatures and pressures part1l--amino acids. *J. Chem. Soc. Faraday Trans.* **93**, 1927–1941 (1997). 10.1039/a608126f 10796027

[31] Dickson, K., Burns, C. & Richardson, J. Determination of the free-energy change for repair of a dna phosphodiester bond. *J. Biol. Chem.* **275**, 15828–15831 (2000). 10748184 10.1074/jbc.M910044199

[32] Petrache, H. I. 5.2 lipid bilayer structure. In Egelman, E. H. (ed.) *Comprehensive Biophys*. 3–15 (2012).

[33] Lynch, M. & Marinov, G. K. The bioenergetic costs of a gene. *Proc. Natl. Acad. Sci. USA* **112**, 15690–15695 (2015). 26575626 10.1073/pnas.1514974112 PMC4697398

[34] Lynch, M. & Marinov, G. K. Membranes, energetics, and evolution across the prokaryote-eukaryote divide. *eLife.* **6**, e20437 (2017). 28300533 10.7554/eLife.20437 PMC5354521

[35] Engelbrecht, K. C., Putonti, C., Koenig, D. W. & Wolfe, A. J. Draft genome sequence of Escherichia coli k-12 (ATCC 29425). *Genome Announc.* **5**, e00574-17 (2017). 28684575 10.1128/genomeA.00574-17 PMC5502856

[36] Jones, B., Stekel, D., Rowe, J. & Fernando, C. Is there a liquid state machine in the bacterium Escherichia coli? in *2007 IEEE Symposium on Artificial Life*, 187–191 (IEEE, 2007).

[37] Tiessen, A., Pérez-Rodríguez, P. & Delaye-Arredondo, L. J. Mathematical modeling and comparison of protein size distribution in different plant, animal, fungal and microbial species reveals a negative correlation between protein size and protein number, thus providing insight into the evolution of proteomes. *BMC Res. Notes* **5**, 85 (2012). 22296664 10.1186/1756-0500-5-85 PMC3296660

[38] Akashi, H. & Gojobori, T. Metabolic efficiency and amino acid composition in the proteomes of Escherichia coli and Bacillus subtilis. *Proc. Natl. Acad. Sci. USA* **99**, 3695–3700 (2002). 11904428 10.1073/pnas.062526999 PMC122586

[39] Prats, R. & de Pedro, M. A. Normal growth and division of Escherichia coli with a reduced amount of murein. *J. Bacteriol.* **171**, 3740–3745 (1989). 2500418 10.1128/jb.171.7.3740-3745.1989 PMC210119

[40] Niebel, B., Leupold, S. & Heinemann, M. An upper limit on gibbs energy dissipation governs cellular metabolism. *Nat. Metab.* **1**, 125–132 (2019). 32694810 10.1038/s42255-018-0006-7

[41] Feist, A. M. & Palsson, B. O. The biomass objective function. *Curr. Opin. Microbiol.* **13**, 344–349 (2010). 20430689 10.1016/j.mib.2010.03.003 PMC2912156

[42] Yuan, H., Cheung, C. Y. M., Hilbers, P. A. J. & van Riel, N. A. W. Flux balance analysis of plant metabolism: The effect of biomass composition and model structure on model predictions. *Front. Plant Sci.* **7**, 1–13 (2016). 27200014 10.3389/fpls.2016.00537 PMC4845513

[43] von Kamp, A. & Klamt, S. Balancing biomass reaction stoichiometry and measured fluxes in flux balance analysis. *Bioinformatics* **39**, btad600 (2023). 10.1093/bioinformatics/btad600 PMC10568370 37758251

[44] Varma, A., Boesch, B. & Palsson, B. Stoichiometric interpretation of Escherichia coli glucose catabolism under various oxygenation rates. *Appl. Environ. Microbiol.* **59**, 2465–2473 (1993). 8368835 10.1128/aem.59.8.2465-2473.1993 PMC182307

[45] Orth, J. D., Thiele, I. & Palsson, B. O. What is flux balance analysis?. *Nat. Biotechnol.* **28**, 245–248 (2010). 20212490 10.1038/nbt.1614 PMC3108565

[46] Monk, J. M. *et al.* iML1515, a knowledgebase that computes escherichia coli traits. *Nat. Biotechnol*. **35**, 904–908 (2017). Number: 10 Publisher: Nature Publishing Group. 10.1038/nbt.3956 PMC6521705 29020004

[47] Doerr, A. Sequencing for carbohydrates. *Nat. Methods* **14**, 1126–1126 (2017). 10.1038/nmeth.4528

[48] Kalwarczyk, T., Tabaka, M. & Holyst, R. Biologistics-diffusion coefficients for complete proteome of Escherichia coli. *Bioinformatics* **28**, 2971–2978 (2012). 22942021 10.1093/bioinformatics/bts537 PMC3496334

[49] Capolupo, L. Single-cell lipidomics reveals the organizing principle of cell fate decision. *Nat. Rev. Mol. Cell Biol.* **24**, 377–377 (2023). 36914763 10.1038/s41580-023-00595-x

[50] Johnson, J. W., Oelkers, E. H. & Helgeson, H. C. SUPCRT92: A software package for calculating the standard molal thermodynamic properties of minerals, gases, aqueous species, and reactions from 1 to 5000 bar and 0 to 1000c. *Comput. Geosci.* **18**, 899–947 (1992). 10.1016/0098-3004(92)90029-Q

[51] Booth, I. The regulation of intracellular pH in bacteria. *Novartis Foundation Symp.* **221**, 19–37 (1999). 10.1002/9780470515631.ch3 10207911

[52] Boron, W. Regulation of intracellular pH. *Adv. Physiol. Educ.* **28**, 160–179 (2004). 15545345 10.1152/advan.00045.2004

[53] Cockell, C. S. *et al.* Sustained and comparative habitability beyond earth. *Nat. Astron*. 1–9 (2023).

[54] Rouphael, Y. *et al.* Reducing energy requirements in future bioregenerative life support systems (BLSSs): Performance and bioactive composition of diverse lettuce genotypes grown under optimal and suboptimal light conditions. *Front. Plant Sci*. **10** (2019). 10.3389/fpls.2019.01305 PMC6831738 31736990

[55] Ra, K., Shiotsu, F., Abe, J. & Morita, S. Biomass yield and nitrogen use efficiency of cellulosic energy crops for ethanol production. *Biomass Bioenergy* **37**, 330–334 (2012). 10.1016/j.biombioe.2011.12.047

[56] Wallace, D. C. Bioenergetics, the origins of complexity, and the ascent of man. *Proc. Natl. Acad. Sci.* **107**, 8947–8953 (2010). 20445102 10.1073/pnas.0914635107 PMC3024017

[57] Xu, C., Hu, S. & Chen, X. Artificial cells: From basic science to applications. *Mater. Today (Kidlington)* **19**, 516–532 (2016). 28077925 10.1016/j.mattod.2016.02.020 PMC5222523

[58] Robinson, K. J. *et al.* Quantifying the extent of amide and peptide bond synthesis across conditions relevant to geologic and planetary environments. *Geochimica et Cosmochimica Acta* **300**, 318–332 (2021). 10.1016/j.gca.2021.01.038

[59] Lever, M. *et al.* Life under extreme energy limitation: A synthesis of laboratory-and field-based investigations. *FEMS Microbiol. Rev.* **39**, 688–728 (2015). 25994609 10.1093/femsre/fuv020

[60] Tijhuis, L., Van Loosdrecht, M. & Heijnen, J. A thermodynamically based correlation for maintenance gibbs energy requirements in aerobic and anaerobic chemotrophic growth. *Biotechnol. Bioeng.* **42**, 509–519 (1993). 18613056 10.1002/bit.260420415

[61] Mavrovouniotis, M. L. Estimation of standard gibbs energy changes of biotransformations. *J. Biol. Chem.* **266**, 14440–14445 (1991). 1860851 10.1016/S0021-9258(18)98705-3

[62] Leal, A. Reaktoro, a unified open-source framework for modeling chemically reactive systems. https://reaktoro.org (2015).

[63] Shock, E., Oelkers, E., Johnson, J., Sverjensky, D. & Helgeson, H. Calculation of the thermodynamic properties of aqueous species at high pressures and temperatures. effective electrostatic radii, dissociation constants and standard partial molal properties to 1000 c and 5 kbar. *J. Chem. Soc. Faraday Trans.* **88**, 803–826 (1992).

[64] Van Meer, G., Voelker, D. & Feigenson, G. Membrane lipids: where they are and how they behave 60. *Nat. Rev. Mol. Cell Biol.* **9**, 112–124 (2008). 18216768 10.1038/nrm2330 PMC2642958

[65] Sohlenkamp, C., López-Lara, I. & Geiger, O. Biosynthesis of phosphatidylcholine in bacteria. *Prog. Lipid Res.* **42**, 115–162 (2003). 12547654 10.1016/S0163-7827(02)00050-4

[66] Conde-Alvarez, R. *et al.* Synthesis of phosphatidylcholine, a typical eukaryotic phospholipid, is necessary for full virulence of the intracellular bacterial parasite brucella abortus. *Cell Microbiol.* **8**, 1322–1335 (2006). 16882035 10.1111/j.1462-5822.2006.00712.x

[67] Henry, C. S., Jankowski, M. D., Broadbelt, L. J. & Hatzimanikatis, V. Genome-scale thermodynamic analysis of Escherichia coli metabolism. *Biophys. J.* **90**, 1453–1461 (2006). 16299075 10.1529/biophysj.105.071720 PMC1367295

[68] Jankowski, M. D., Henry, C. S., Broadbelt, L. J. & Hatzimanikatis, V. Group contribution method for thermodynamic analysis of complex metabolic networks. *Biophys. J.* **95**, 1487–1499 (2008). 18645197 10.1529/biophysj.107.124784 PMC2479599

[69] Geopig slop files [slop07]. *Zenodo*, v1. 10.5281/zenodo.2630820 (2019). 10.5281/zenodo.2630820

