# Supplementary material for: The minimum energy required to build a cell
Source: Sci Rep. 2024 Mar 4;14:5267. doi: 10.1038/s41598-024-54303-6 (PMC11306549; doi:10.1038/s41598-024-54303-6)
Supplement: Supplementary file 1 — Supplementary Information 1. [file 41598_2024_54303_MOESM1_ESM.pdf]

Supplementary information for: The Minimum Energy Required to Build a Cell

E. Ortega-Arzola, P. M. Higgins and C. S. Cockell

January 17, 2024

This supplementary information document provides additional details and data supporting the main findings of the study. It elaborates on some specific equations and parameters used in the model, as well as a more detailed description of the theoretical and experimental methods used to calculate the energy necessary to synthesise a cell.

The document also provides a comparison of the energy required to synthesise different cell types (for *Saccharomyces cerevisiae*, and an average mammalian cell) under different conditions, such as variations in temperature.

Overall, the supplementary information document provides a more comprehensive and technical overview of the research presented in the main paper, including additional data and analyses that support the conclusions of the study.

Composition, Mass fractions, concentrations and main properties of building blocks per cell type

Table S1. Cell composition by fraction of mass and type. Using a combination of theoretic and experimental data from the literature, we calculated the grams of DNA, RNA, proteins, lipids, glycans and menabolites. This information was used to adjust the biomolecule concentrations in the virtual cell calculations.

Concentration according to fraction of mass					
	**JCVI-syn3A**	***E coli***	***S cerevisiae***	**Mammalian**	
**Single cell wet weight [A,B,C]**	4.08E-14	9.5E-13	7.92E-11	3.50E-09	**g**
**Water content [D,E,F]**	72	70	80	70	**%***
	2.94E-17	6.65E-16	6.34E-14	2.45E-12	**L**
**Single cell dry weight [G,H,I]**	1.02E-14	2.85E-13	1.584E-11	1.05E-09	**g**
**Cells in 1 dry gram**	9.80E+13	3.51E+12	6.31E+10	9.52E+08	**Cells**
**Volume per cell [J,K,L]**	1	1	110	3000	**um3**
**Cell density**	1.1	1.105	1.1126	1.05	**g/ml**
**From fraction of dry mass per cell [M - T]**					
**DNA**	**5.5%**	**3.1%**	**0.3%**	**0.9%**	**%****
**Genome mass**	5.61E-16	8.91E-15	5.15E-14	9.33E-12	**g**
**Genome size [U]**	5.43E+05	4.60E+06	1.21E+07	6.40E+09	**bp**
**Number of Genes**	493				
**Genome weight**	355,122,000.00	3.01E+09	7.89E+09	4.19E+12	**g/mol**
**Moles of DNA**	1.58E-24	2.96E-24	6.52E-24	2.23E-24	**mol**
**Genome molarity**	5.38E-08	4.46E-09	1.03E-10	9.10E-13	**mol/L±**
**RNA**	**16.3%**	**19.3%**	**8.3%**	**3.8%**	**%****
**RNA mass**	1.66E-15	5.49E-14	1.32E-12	3.97E-11	**g**
**Mean RNA length [V]**	54	1000	1250	2000	**nts**
**Number of RNAs**	5.45E+04	9.73E+04	1.87E+06	3.52E+07	
**Number of RNA nucleotides**	2.94E+06	9.73E+07	2.34E+09	7.04E+10	
**Transcriptome weight**	9.99E+08	3.30E+10	7.95E+11	2.39E+13	**Da**
**Moles of RNA**	1.66E-24	1.66E-24	1.66E-24	1.66E-24	**mol**
**Transcriptome molarity**	5.65E-08	2.50E-09	2.62E-11	6.78E-13	**mol/L±**
**Proteins**	**55%**	**55%**	**43%**	**60%**	**%****
**Proteome mass**	5.58E-15	1.57E-13	6.82E-12	6.30E-10	**g**
**Mean protein length [W]**	385	320	501	430	**AAs**
**Mean protein weight**	42350.00	35200.00	55110.00	47300.00	**g/mol**
**Number of AAs**	3.05E+07	8.58E+08	3.73E+10	3.45E+12	**g**
**Number of proteins**	7.93E+04	2.68E+06	7.45E+07	8.02E+09	
**Moles of protein**	1.32E-19	4.45E-18	1.24E-16	1.33E-14	**mol**
**Proteome concentration**	4.48E-03	6.70E-03	1.95E-03	5.44E-03	**mol/L±**
**Lipids**	**17.5%**	**9.3%**	**4.8%**	**15.4%**	**%****
**Membrane mass**	1.79E-15	2.65E-14	7.66E-13	1.62E-10	**g**
**Number of phospholipids**	3.17E+06	4.70E+07	1.36E+09	2.88E+11	
**Membrane weight**	2.34E+09	3.48E+10	1.00E+12	2.13E+14	**Da**
**Moles of phospholipids**	7.62E-25	7.62E-25	7.62E-25	7.62E-25	**mol**
**Phospholipids concentration**	2.59E-08	1.15E-09	1.20E-11	3.11E-13	**mol/L**
**Carbohydrates**	**10%**	**10%**	**36%**	**7%**	**%****
**Total Carbohydrates mass**	1.02E-15	2.91E-14	5.65E-12	7.00E-11	**g**
**Carbohydrates concentration**	3.47E+01	4.38E+01	8.93E+01	2.86E+01	**g/L**
**Metabolites and ions**	**5.9%**	**5.6%**	**9.5%**	**13.2%**	**%****
Total Mass	6.03E-16	1.61E-14	1.50E-12	1.39E-10	**g**
Concentration	2.05E+01	2.41E+01	2.36E+01	5.66E+01	**g/L**

Values for E. coli grown at 37 °C in aerobic glucose containing minimal medium at a doubling time of 40 minutes. Values represent an average of reported values for various growth conditions. Values for S. cerevisiae grown at 30 °C in aerobic 0.5% glucose containing minimal medium at a doubling time of 160 minutes. Values represent an average of reported values for various growth conditions. *Percentage of total cell weight. **Percentage of dry mass per cell. ±Volume of intracellular liquid per cell. Calculated using data from: **A**;[25, 21], **B**;[26], **C**;[27], **D**;[9, 14], **E**;[9, 14], **F**;[9, 30], **G**;[25], **H**;[31], **I**;[18], **J**;[17, 28], **K**;[26, 32, 7], **L**;[**milocell**, 4, 19], **M - T**; [**Nelson2004Lehninger**, **lengeler_biology_2009**, **milocell**, **gombert_network_2001**, **Encyclopaedia_Britannica**, 1, 21, 9, 29] **U**;[**massie_molecular_1965**], **V**;[**miura_absolute_2008**, 16], **W**;[**milocell**, **warringer_evolutionary_2006**, 2],

Table S2. Properties and characteristics of the protein building blocks used in our model

Protein building blocks												
**Name**	**Molecular structure**	**Weight (Da)**	**ΔG_f° (kJ mol)**	**$\Delta G_f^{'\circ}$ (kJ mol)**	***E. coli* Absolute intracellular concentration (mol/L)**	***S. cerevisiae* Absolute intracellular concentration (mol/L)**	**Mammalian cell (iBMK) Absolute intracellular concentration (mol/L)**	**JCVI-syn3A Absolute intracellular concentration (mol/L)**	**Name**	**Molecular structure**	**Weight (Da)**	**ΔG_f° (kJ mol)**
		**(Da)**	**(kJ mol)**	**(kJ mol)**	**(mol/L)**	**(mol/L)**	**(mol/L)**	**(mol/L)**			**(Da)**	**(kJ mol)**
	**SUPCRT92** [15]				**Bennett et al. 2009 [3] & Park et al. 2016 [23]**			**Breuer et al. 2019 [5]**	**SUPCRT92** [15]			
Amino acids									**Backbone**			
**Ala**	C3H7NO2	89	-371.56	-92.15	2.81E-04	8.12E-05	4.23E-05	9.526E-03	**Ala [R]**	C3H5NO	71.08	-26.89
**ARG**	C6H14N4O2	174	-239.94	318.86	5.69E-04	2.18E-02	2.55E-04	2.728E-03	**ARG [R]**	C6H12N4O	156.19	104.72
**ASN**	C4H8N2O3	132	-525.01	-205.7	5.11E-04	5.69E-03	1.56E-04	6.897E-03	**ASN [R]**	C4H6N2O2	114.11	-180.35
**ASP**	C4H7NO4	133	-721.75	-442.35	4.23E-03	6.29E-03	1.49E-02	5.984E-03	**ASP [R]**	C4H5NO3	115.09	-377.09
**CYS**	C3H7NO2S	121	-336.07	-56.67	-	-	8.40E-05	6.831E-04	**CYS [R]**	C3H5NOS	103.15	8.59
**GLN**	C5H10N2O3	148	-724	-364.77	3.81E-03	3.55E-02	1.62E-02	3.608E-03	**GLN [R]**	C5H8N2O2	128.13	-379.34
**GLU**	C5H9NO4	147	-528.32	-129.17	9.60E-02	3.91E-02	4.36E-02	6.325E-03	**GLU [R]**	C5H7NO3	129.12	-183.65
**HIS**	C6H9N3O2	155	-202.55	156.68	6.76E-05	-	4.10E-04	1.331E-03	**HIS [R]**	C6H7N3O	137.14	142.11
**ILE**	C6H13NO2	131	-343.01	175.87	1.52E-04	3.53E-04	1.66E-03	9.625E-03	**ILE [R]**	C6H11NO	113.16	1.65
**LEU**	C6H13NO2	131	-352.26	166.63	1.52E-04	3.53E-04	1.66E-03	9.383E-03	**LEU [R]**	C6H11NO	113.16	-7.6
**LYS**	C6H14N2O2	146	-337.53	221.27	4.05E-04	5.16E-03	5.06E-04	9.977E-03	**LYS [R]**	C6H12N2O	128.18	7.13
**MET**	C5H11NO2S	149	-502.54	-63.48	1.45E-04	1.91E-04	6.19E-04	1.947E-03	**MET [R]**	C5H9NOS	131.2	-157.88
**PHE**	C9H11NO2	165	-206.78	232.28	1.82E-05	2.73E-04	7.97E-04	3.256E-03	**PHE [R]**	C9H9NO	147.18	137.88
**PRO**	C5H9NO2	115	-307.74	51.49	3.85E-04	1.36E-03	1.23E-03	3.322E-03	**PRO [R]**	C5H7NO	97.12	36.92
**SER**	C3H7NO3	105	-518.49	-239.09	1.13E-03	3.87E-03	4.86E-03	7.381E-03	**SER [R]**	C3H5NO2	87.08	-173.83
**THR**	C4H9NO3	119	-501.34	-142.11	1.26E-03	6.69E-03	6.69E-03	6.600E-03	**THR [R]**	C4H7NO2	101.11	-156.68
**TRP**	C11H12N2O2	204	-112.18	366.8	1.21E-05	5.55E-05	1.80E-04	4.565E-04	**TRP [R]**	C11H10N2O	186.22	232.48
**TYR**	C9H11NO3	181	-384.05	55.01	2.89E-05	2.48E-04	8.88E-04	2.508E-03	**TYR [R]**	C9H9NO2	163.18	-39.39
**VAL**	C5H11NO2	117	-356.99	82.07	4.02E-03	2.50E-03	1.44E-03	7.777E-03	**VAL [R]**	C5H9NO	99.13	-12.33
**GLY**	C2H5NO2	75	-380.51	-180.94	-	-	3.71E-03	9.922E-03	**GLY [R]**	C2H3NO	57.05	-35.85
**GLY link**	-	57	-120.15	-0.4	-	-	-		**GLY link [R]**	-	-	224.51
									**AABB**	H2NCHCOOH	74.05	-344.66
									**PBB**	HNCHCO	56.04	-84.3

We examined both thermodynamic and biological standard ΔG_f° for the building blocks [22] to ensure consistency in results between them. Each standard estimated the same ΔG_r, potentially due to the lack of H^+ and our assumption that ionic strength is close to zero [13]. The ΔG_f° of both standards required for protein polymerisation is a similar order of magnitude to other estimates [12, 2].

Table S3. Properties and characteristics of the nucleic acid building blocks used in our model. Nucleotides ion are those with a hydrogen removed from the phosphate and one OH group removed from the 3' to be stacked on top of each with help of the ester energy other to create a chain. Concentrations for the nucleotides or the nucleotides ion are assumed to be the same.

Nucleic Acid Building Blocks							
	SUPCRT92 [15]			Bennet et al. [3] and Park et al. [23]			Breuer [5]
	**Molecular formula**	**Weight(Da)**	$\Delta G_f^{'\circ}$ **(kJ mol)**	**Absolute intracellular concentration in *E. coli* (mol/L)**	**Absolute intracellular concentration in *S. cerevisiae* (mol/L)**	**Absolute intracellular concentration in a mammalian cell (mol/L)**	**Absolute intracellular concentration in JCVI-syn3A (mol/L)**
**Phosphate backbone (PBB)**							
**Orthophosphate**	PO4-	96.98	-1018.83	2.39E-02	4.93E-02	5.83E-03	-
**2'-deoxyribose (aq)**	C5H10O4	134.06	-205.12	3.03E-04	-	-	-
**2'-ribose (aq)**	C5H10O5	150.1299	-352.84	-	1.52E-04	7.83E-05	-
Bases							
**Adenine (aq)**	C5H5N5	135.12	512.44	1.47E-06	-	-	-
**Cytosine (aq)**	C4H5N3O	111.04	164.41	2.59E-06	-	-	-
**Guanine (aq)**	C5H5N5O	151.12	269.48	-	-	1.88E-04	-
**Thymine (aq)**	C5H6N2O2	126.11	-17.02	-	3.45E-06	2.64E-06	-
**Uracil (aq)**	C4H4N2O2	112.08	-90.24	2.10E-03	-	-	-
**Nucleosides**							
DNA							
**Deoxyadenosine**	C10H13N5O3	251.24	472.11	2.82E-06	-	-	-
**Deoxycytidine**	C9H13N3O4	227.21	114.55	-	-	-	-
**Deoxyguanosine**	C10H13N5O4	267.24	219.61	5.22E-07	-	-	-
**Deoxythymidine**	C10H14N2O5	242.2	-66.88	-	-	-	-
RNA							
**Adenosine**	C10H13N5O4	267.24	324.39	1.31E-07	-	-	-
**Cytidine**	C9H13N3O5	243.21	-25.72	1.41E-05	-	-	-
**Guanosine**	C10H13N5O5	283.24	77.24	1.62E-06	-	1.35E-06	-
**Uridine**	C9H12N2O6	244.2	-280.37	2.09E-03	-	-	-
**Nucleotides**							-
DNA							
**dAMP**	C10H14N5O6P	331.22	-406.58	8.84E-06	6.27E-05	1.68E-05	6.27E-05
**dCMP**	C9H14N3O7P	307.2	-764.12	-	-	3.71E-05	4.18E-05
**dGMP**	C10H14N5O7P	347.22	-659.06	5.07E-05	-	-	2.42E-05
**dTMP**	C10H15N2O8P	322.21	-945.56	-	-	1.18E-05	7.48E-05
RNA							
**AMP**	C10H14N5O7P	347.22	-550.56	2.81E-04	8.12E-05	4.23E-05	3.62E-03
**CMP**	C9H14N3O8P	323.2	-904.23	3.60E-04	5.18E-06	1.18E-05	1.21E-03
**GMP**	C10H14N5O8P	363.22	-801.47	2.37E-05	1.02E-05	1.81E-05	2.41E-03
**UMP**	C9H13N2O9P	324.18	-1158.89	-	1.45E-05	1.45E-05	2.41E-03
**Nucleotides ion**							
DNA							
**dAMP-2 (ion)**	C10H12N5O5P-2	313.21	-408.47	8.84E-06	-	1.68E-05	-
**dCMP-2 (ion)**	C9H12N3O6P-2	289.18	-764.77	-	-	3.71E-05	-
**dGMP-2 (ion)**	C10H12N5O6P-2	329.21	-660.96	5.07E-05	-	-	-
**dTMP-2 (ion)**	C10H13N2O7P-2	304.19	-947.46	-	-	1.18E-05	-
RNA							
**AMP-2 (ion)**	C10H12N5O6P-2	329.21	-556.18	2.81E-04	8.12E-05	4.23E-05	-
**CMP-2 (ion)**	C9H12N5O7P-2	333.19	-906.31	3.60E-04	5.18E-06	1.18E-05	-
**GMP-2 (ion)**	C10H12N5O7P-2	345.2	-803.33	2.37E-05	1.02E-05	1.81E-05	-
**UMP-2 (ion)**	C9H11N2O8P-2	306.17	-1160.96	-	1.45E-05	1.45E-05	-
**Metabolites**							
**Glucose-6-phosphate**	C6H11O9P	258.12	-1265.58	7.88E-03	5.31E-03	6.75E-04	-
**ATP**	C10H16N5O13P3	507.18	-2200.13	9.63E-03	1.93E-03	4.67E-03	-
**ADP**	C10H15N5O10P2	427.2	-1370.82	5.55E-04	4.88E-04	5.69E-04	-
**Glutamine**	C5H10N2O3	146.14	-129.17	3.81E-03	3.55E-02	1.62E-02	-
**O2**	O2	31.99	16.55	-	-	-	-
**CO2**	CO2	44	-385.95	7.52E-05	8.16E-05	7.63E-03	-

Stoichiometry for POPC building blocks

Table S4. Stoichiometry to synthesise POPC building blocks from metabolites found in the database. Highlighted in pink are the key metabolites to synthesise POPC while in green are the metabolites found in the SUPCRT92 database necessary to calculate ΔG_r.

Group contribution
Source: MetaCyc

**POPC**								
Building block	Olaeate	Palmitate	Choline	Phosphate	Glycerol		**POPC**	Water
Formula	C18H34O2	C16H32O2	C5H14NO	H2O4P	C3H8O3		C42H82NO8P	H2O
Stoichiometry	1	1	1	1	1	=	1	4
Formation energy (KJ/mol)	2638.16	2345.18	386.974	-1050.42	177.455		5126.709	-157.34
**Choline**								
Building block	**Serine**	**ATP**	Water	=	Choline	ADP	Phosphate	Proton
Formula	C3H7NO3	C10H16N5O13P3	H2O		C5H14NO	C10H15N5O10P2	PO4	H
Stoichiometry	3	7.4	0.2		1	7.8	6.6	8.8
Formation energy (KJ/mol)	-239.09	-2200.13	-157.34		386.974	-1370.82	-1018.83	0
**Glycerol**								
Building block	**Glucose**	ATP	**Oxygen**	=	Glycerol	ADP	Phosphate	
Formula	C6H12O6	C10H16N5O13P3	O2		C3H8O3	C10H15N5O10P2	PO4	
Stoichiometry	0.5	2	1		1	2	2	
Formation energy (KJ/mol)	-436.27	-2200.13	16.55		177.455	-1370.82	-1018.83	
**Glyceraldehyde 3 phosphate**								
Building block	Glucose	ATP		=	Glyceraldehyde 3-phosphate	ADP		
Formula	C6H12O6	C10H16N5O13P3			C3H7O6P	C10H15N5O10P2		
Stoichiometry	0.5	1			1	1		
Formation energy (KJ/mol)	-436.27	-2200.13			-1047.445	-1370.82		
**Pyruvate**								
Building block	Glyceraldehyde 3-phosphate	ADP	Phosphate	=	Pyruvate	ATP	Water	
Formula	C3H7O6P	C10H15N5O10P2	PO4		C3H4O3	C10H16N5O13P3	H2O	
Stoichiometry	1	2	1		1	2	1	
Formation energy (KJ/mol)	-1047.44	-1370.82	-1018.83		-250.31	-2200.13	-157.34	
**Palmitate**								
Building block	Pyruvate	Malonate		=	Palmitate	Carbon dioxyde	Water	
Formula	C3H4O3	C3H2O4			C16H32O2	CO2	H2O	
Stoichiometry	8	2			1	14	2	
Formation energy (KJ/mol)	-250.31	-685.16			2345.18	-385.95	-157.34	
**Oleate**								
Building block	Pyruvate	Malonate		=	Oleate	Carbon dioxyde	Water	
Formula	C3H4O3	C3H2O4			C18H34O2	CO2	H2O	
Stoichiometry	9	2			1	15	3	
Formation energy (KJ/mol)	-250.31	-685.16			2638.16	-385.95	-157.34	

The building blocks of POPC are oleate, palmitate, choline, glycerol, and phosphate, from which only phosphate is available in slop07. We used ATP and glucose to make pyruvate, following the synthesis pathway of glycolysis and glycerol [10, 20, 8]. Malonate is included in the slop07 database and was used with pyruvate to build palmitate and oleate [6].

Supplemental Figures

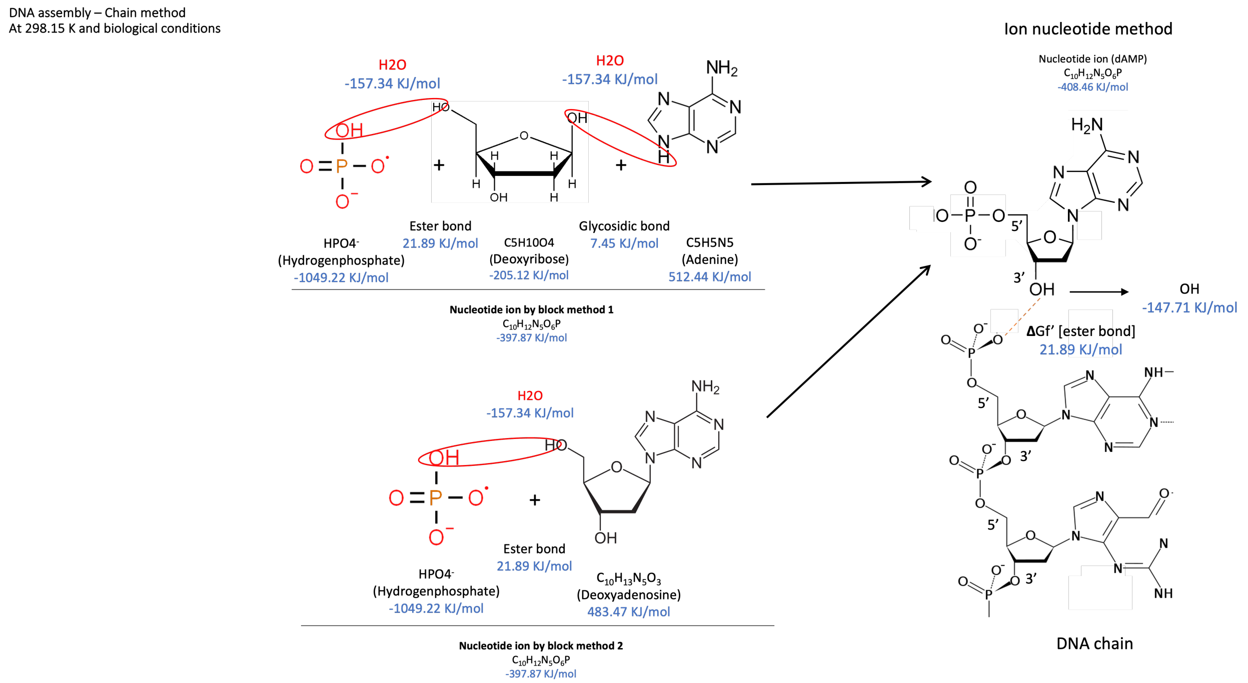

Fig. S1. Block Methods: The ion nucleotides of DNA chains were broken down twice to confirm the GCA efficiency. We refer to the 'chain method' as the standard energetic calculation, other two methods were assessed in this work. First, we broke down the ion nucleotide into phosphate, sugar, base and its respective bonds ('block 2 method'), and secondly into a phosphate, nucleoside and bonds ('block 2 method'). the calculations were done using information available in slop07.

To test the accuracy of the GCA, we broke down the nucleotides of *E. coli*'s genome in three different ways when calculating the cost of synthesis (Supplementary **Fig. S1.** and methods section). The three methods have similar energetic requirements, although some discrepancy can be seen between the chain method and both block methods (0.12 kJ/g for the chain method and 0.152 kJ/g for both block methods).

A similar small energetic discrepancy between the chain method and both block methods can be seen when building the nucleotides from smaller components (GCA) to calculate the ΔG_f° of each nucleotide. When taking dAMP as an example (Supplementary material; **Fig. S1**), and adding together the values of the three different building blocks (**Block method 1:** phosphate + deoxyribose + adenine; **Fig. S1**) plus their respective bond energies, we obtain a value of -397.87 kJ/mol which differs by around 10 kJ/mol from the dAMP ion (-408.46 kJ/mol) used in the chain method. Similarly, when building the nucleotide from two building blocks (**Block method 2:** phosphate + deoxyadenosine), we obtain the same result (-397.87 kJ/mol) as with block method 1. This suggests a potential intramolecular interaction being ignored or a small error in measuring the smaller building blocks. Due to the slight differences between the chain method and both block methods, we determined to use the chain method as the standard for the ΔG_r calculations of nucleic acids.

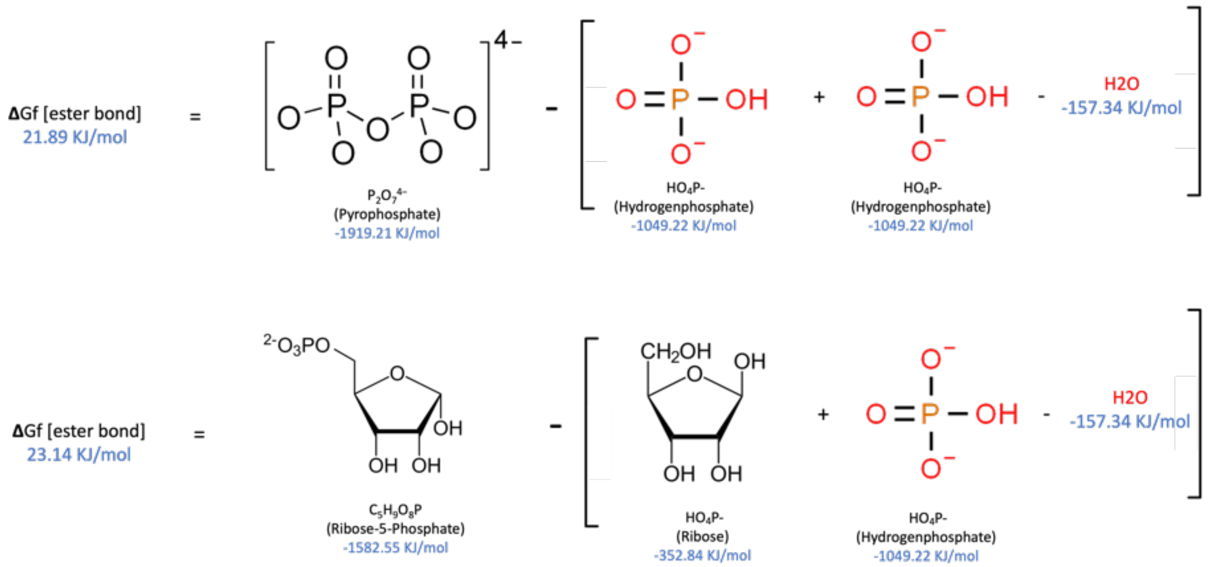

Fig. S2. Energy to build an ester bond using the GCA

Because the energetic contribution of intramolecular bonds could significantly affect the overall biomolecule's ΔG_f°, we calculated the energetic requirement of the critical DNA-binding ester bond using the GCA. This involved subtracting the building blocks' ΔG_f° of relatively simple structures with well-documented thermodynamic data. For instance, we removed the energy of the ribose and a phosphate to ribose-5-phosphate and obtained a value of 23.14 kJ/mol (Supplementary **Fig. S2**) which approximately matches the hydrolysis value obtained experimentally by Dickson, Burns & Richardson [11] (−22.175 kJ/mol) at 25 C and pH 7.

Since this value was calculated from the bond hydrolysis, the formation energy is approximately the inverse of this (22.17 kJ / mol). Similarly, we obtained the glycosidic bond's ΔG_f° (0.304 kJ/mol)

We assessed the energetic requirements of protein synthesis with two sets of standards. The biological and thermodynamic standard ΔG_f° provided the same results across the entire temperature range, indicating they are reliable. Adding more variables to our model could change this, and yield other important insights. For instance, pH under physiological conditions can be different for different organisms and change with temperature [24]. In the future, adding this variable into the model could give further insights into how temperature affects synthesis cost.

We assessed different building methods for nucleic acids to calculate a DNA chain's ΔG_f° and examine the GCA's effectiveness. To do this, we broke down every nucleotide of an input sequence into 5 (block method 1) or 3 (block method 2) fractions (Supplementary **Fig. S1**) and used them in the original equation [7] for nucleic acids yielding similar standard ΔG_f° for the DNA chain. Since block methods 1 and 2 have identical values, we can determine that the GCA is a suitable estimate even for intramolecular interactions. At the same time, the GCA methods described here could provide a quantitative basis to inform future studies about possible interactions between the smaller building blocks that are not currently considered in models like this.

Despite the predictions of both block methods being similar, some discrepancies can be seen with the chain method. In some cases, this discrepancy could help characterise the intramolecular bond energies between the smaller building blocks and the nucleotide (e.g. the ester and glycosidic bonds). This is important to understanding the intramolecular interactions as part of the building blocks and how they change with the temperature. More details about this step can be seen in **Fig. S4**.

In this work, we present different models for estimating the energy required to build the biomactomolecules in a cell from their metabolic building blocks. The models are contained in a tool called Syncell that can be integrated into other models. Using omics data and information about the internal cell composition the model can estimate the energy required to build any well-sequenced species at a given temperature.

References

[1] B. Alberts, ed. *Molecular biology of the cell.* 4th ed. New York: Garland Science, 2002. ISBN: 978-0-8153-3218-3 978-0-8153-4072-0.

[2] J. Amend, D. LaRowe, T. McCollom, et al. “The energetics of organic synthesis inside and outside the cell”. In: *Philos Trans R Soc B Biol Sci* 368 (2013), p. 20120255.

[3] B. Bennett, E. Kimball, M. Gao, et al. “Absolute Metabolite Concentrations and Implied Enzyme Active Site Occupancy in Escherichia coli”. In: *Nat Chem Biol* 5 (8 2009), pp. 593–599.

[4] E. Bianconi, A. Piovesan, F. Facchin, et al. “An estimation of the number of cells in the human body”. In: *Ann Hum Biol* 40 (6 2013), pp. 463–471.

[5] M. Breuer, T. M. Earnest, C. Merryman, et al. “Essential metabolism for a minimal cell”. In: *eLife* 8 (2019). Ed. by Z. Nikoloski and N. Barkai. Publisher: eLife Sciences Publications, Ltd, e36842. DOI: 10.7554/eLife.36842.

[6] R. Caspi, T. Altman, R. Billington, et al. “The MetaCyc database of metabolic pathways and enzymes and the BioCyc collection of Pathway/Genome Databases”. In: *Nucleic Acids Research* 42 (D1 2014), pp. D459–D471.

[7] Y.-H. Chan and W. Marshall. “Organelle Size Scaling of the Budding Yeast Vacuole Is Tuned by Membrane Trafficking Rates”. In: *Biophys J* 106 (9 2014), pp. 1986–1996.

[8] G. Cronwright, J. Rohwer, and B. Prior. “Metabolic Control Analysis of Glycerol Synthesis in Saccharomyces cerevisiae”. In: *Appl Environ Microbiol* 68 (9 2002), pp. 4448–4456.

[9] F. Delgado, N. Cermak, V. Hecht, et al. “Intracellular Water Exchange for Measuring the Dry Mass, Water Mass and Changes in Chemical Composition of Living Cells”. In: *PLOS ONE* 8 (7 2013), e67590.

[10] E. Deplazes, D. Poger, B. Cornell, et al. “The effect of hydronium ions on the structure of phospholipid membranes”. In: *Phys Chem Chem Phys* 20 (1 2017), pp. 357–366.

[11] K. Dickson, C. Burns, and J. Richardson. “Determination of the Free-Energy Change for Repair of a DNA Phosphodiester Bond”. In: *J Biol Chem* 275 (21 2000), pp. 15828–15831.

[12] P. M. Higgins and C. S. Cockell. “A bioenergetic model to predict habitability, biomass and biosignatures in astrobiology and extreme conditions”. In: *J R Soc Interface* 17 (171 2020), p. 20200588.

[13] P. M. Higgins. “"Modelling extraterrestrial habitability, biomass and biosignatures through the bioenergetic lens"”. PhD thesis. Edinburgh, UK: University of Edinburgh, 2022.

[14] P. Illmer, C. Erlebach, and F. Schinner. “A practicable and accurate method to differentiate between intraand extracellular water of microbial cells”. In: *FEMS Microbiol Lett* 178 (1 1999), pp. 135–139.

[15] J. W. Johnson, E. H. Oelkers, and H. C. Helgeson. “SUPCRT92: A software package for calculating the standard molal thermodynamic properties of minerals, gases, aqueous species, and reactions from 1 to 5000 bar and 0 to 1000°C”. In: *Computers & Geosciences* 18.7 (1992), pp. 899–947.

[16] B. Jones, D. Stekel, J. Rowe, et al. “Is there a Liquid State Machine in the Bacterium Escherichia Coli?” In: *2007 IEEE Symposium on Artificial Life.* 2007 IEEE Symposium on Artificial Life. Honolulu, HI, USA: IEEE, 2007, pp. 187–191. ISBN: 978-1-4244-0701-9.

[17] H. Kubitschek and J. Friske. “Determination of bacterial cell volume with the Coulter Counter”. In: *J Bacteriol* 168 (3 1986), pp. 1466–1467.

[18] B. Łabędź, A. Wańczyk, and Z. Rajfur. “Precise mass determination of single cell with cantilever-based microbiosensor system”. In: *PLOS ONE* 12 (11 2017), e0188388.

[19] K. Luby-Phelps. “Cytoarchitecture and Physical Properties of Cytoplasm: Volume, Viscosity, Diffusion, Intracellular Surface Area”. en. In: *International Review of Cytology.* Ed. by H. Walter, D. E. Brooks, and P. A. Srere. Vol. 192. Microcompartmentation and Phase Separation in Cytoplasm. Academic Press, 1999, pp. 189–221.

[20] S. Maloy and K. Hughes. *Brenner's Encyclopedia of Genetics.* Elsevier Science, 2013. ISBN: 978-0-08-096156-9.

[21] F. C. Neidhardt, ed. *Escherichia coli and Salmonella typhimurium: cellular and molecular biology*. en. Washington, D.C: American Society for Microbiology, 1987. ISBN: 978-0-914826-89-7 978-0-914826-85-9.

[22] E. Noor, H. Haraldsdóttir, R. Milo, et al. "Consistent Estimation of Gibbs Energy Using Component Contributions". In: *PLOS Comput Biol* 9 (7 2013), e1003098.

[23] J. Park, S. Rubin, Y.-F. Xu, et al. "Metabolite concentrations, fluxes, and free energies imply efficient enzyme usage". In: *Nat Chem Biol* 12 (7 2016), pp. 482–489.

[24] R. Reeves. "Temperature-induced changes in blood acid-base status: pH and PCO2 in a binary buffer". In: *J Appl Physiol* 40 (5 1976), pp. 752–761.

[25] T. Sajed, A. Marcu, M. Ramirez, et al. "ECMDB 2.0: A richer resource for understanding the biochemistry of E. coli". In: *Nucleic Acids Res* 44 (2016), pp. D495–501.

[26] F. Sherman. "Getting started with yeast". en. In: *Methods in Enzymology*. Ed. by C. Guthrie and G. R. Fink. Vol. 350. Guide to Yeast Genetics and Molecular and Cell Biology - Part B. Academic Press, 2002, pp. 3–41.

[27] C. Sims and N. Allbritton. "Analysis of single mammalian cells on-chip. Lab Chip". In: 7 (2007), pp. 423–440.

[28] B. Volkmer and M. Heinemann. "Condition-Dependent Cell Volume and Concentration of Escherichia coli to Facilitate Data Conversion for Systems Biology Modeling". In: *PLOS ONE* 6 (7 2011), e23126.

[29] E. Yamada and V. Sgarbieri. "Yeast (Saccharomyces cerevisiae) Protein Concentrate: Preparation, Chemical Composition, and Nutritional and Functional Properties". In: *J Agric Food Chem* 53 (10 2005), pp. 3931–3936.

[30] T. Yamaguchi, C. Muraiso, I. Furuno-Fukushi, et al. "Water Content in Cultured Mammalian Cells for Dosimetry of Beta-rays from Tritiated Water". In: *J Radiat Res* 31 (4 1990), pp. 333–339.

[31] P. Yen Yap and D. Trau. *Direct Yeast Cel Count at OD600*. en. AN 101. Singapore: Tip Biosystems Pte Ltd, 2019, pp. 1–3.

[32] M. Zakhartsev and M. Reuss. "Cell size and morphological properties of yeast Saccharomyces cerevisiae in relation to growth temperature". In: *FEMS Yeast Res* 18 (6 2018).
